# Fungi are more transient than bacteria in caterpillar gut microbiomes

**DOI:** 10.1038/s41598-022-19855-5

**Published:** 2022-09-16

**Authors:** Martin Šigut, Petr Pyszko, Hana Šigutová, Denisa Višňovská, Martin Kostovčík, Nela Kotásková, Ondřej Dorňák, Miroslav Kolařík, Pavel Drozd

**Affiliations:** 1 Department of Biology and Ecology, Faculty of Science, University of Ostrava, Chittussiho 10, 710 00 Ostrava, Czech Republic; 2 Institute of Microbiology, Academy of Sciences of the Czech Republic, Vídeňská 1083, 142 20 Prague, Czech Republic

**Keywords:** Microbial ecology, Entomology, Ecology, Plant ecology

## Abstract

Despite an increasing number of studies on caterpillar (Insecta: Lepidoptera) gut microbiota, bacteria have been emphasized more than fungi. Therefore, we lack data on whether fungal microbiota is resident or transient and shaped by factors similar to those of bacteria. We sampled nine polyphagous caterpillar species from several tree species at multiple sites to determine the factors shaping leaf and gut bacterial and fungal microbiota as well as the extent to which caterpillars acquire microbiota from their diet. We performed 16S and ITS2 DNA metabarcoding of the leaves and guts to determine the composition and richness of the respective microbiota. While spatial variables shaped the bacterial and fungal microbiota of the leaves, they only affected fungi in the guts, whereas the bacteria were shaped primarily by caterpillar species, with some species harboring more specific bacterial consortia. Leaf and gut microbiota significantly differed; in bacteria, this difference was more pronounced. The quantitative similarity between leaves and guts significantly differed among caterpillar species in bacteria but not fungi, suggesting that some species have more transient bacterial microbiota. Our results suggest the complexity of the factors shaping the gut microbiota, while highlighting interspecific differences in microbiota residency within the same insect functional group.

## Introduction

Interactions between insect herbivores and host plants are among the most important ecological associations on Earth^1^. The role of mediating such interactions is played by associated gut microbiota (bacteria, archaea, fungi, protozoa, and viruses)^2,3^. These microorganisms may, among other roles, provide their herbivore hosts with nutrients; aid in the digestion and detoxification of plant tissues; synthesize pheromones; modulate immune responses and communication; govern reproduction; and provide protection against pathogens, predators, and parasitoids^4–6^. Lepidoptera are one of the largest insect herbivore orders and their evolutionary success may depend on their beneficial relationship with microorganisms^5,7^.

Living leaves harbor abundant epiphytic (epiphytes)^8^ and endophytic (endophytes)^9^ microbial communities. Leaf–microbiota relationships range from being clearly negative for the plant^10^ to strongly positive^11^. The leaf microbiota is diverse with bacteria and especially fungi playing the most prominent roles^12,13^, providing interactive potential for leaf consumers^14^. However, the caterpillar midgut is a hostile environment for diet-derived microbes because of its simple tube-like structure, extreme alkalinity (pH 8–12), and high level of plant secondary metabolite content (e.g., allelochemicals) from ingested plant tissues^5,15,16^. Species-specific digestive enzymes are adapted to the gut physiochemical conditions. Therefore, they may act as filters for specific gut microbial communities^5,17^.

There is an ongoing debate around the residency and ecological role of caterpillar gut microbiota^18^. Transiency is supported by the fact that diet best explains the dissimilarity in the microbiomes^19,20^ and that gut changes during the life cycle may prevent the establishment of specific assemblages^21^. Residency is supported by microbiota removal potentially reducing caterpillar fitness^22^. Certain bacterial populations may persist throughout the life cycle, despite extensive gut changes during pupation and metamorphosis^19,23^. Despite the absence of specialized gut structures and rapid food transition^24^, bacteria may form a biofilm, suggesting that they may have an ability to colonize the gut^25^. Therefore, the caterpillar microbiome is likely a multilayer system comprising core taxa and a more flexible non-core microbiome^26,27^ which has a controversial functional role.

Caterpillar gut microbiomes are dynamic and variable with differences in community composition mainly depending on the host phylogeny, life stage, physiological environment, and the diet^4,7,16,19,28–30^. In contrast, the microbiota of the diet, which predominantly comprises leaves, depends on the identity of the plant. Plant species differ in their capacity to harbor microbial communities^12,13,31^. Nutrient availability varies in space and time^32^ depending on environmental factors and the associated physiological activity and productivity^10,33^. Therefore, we can expect host-interspecific and spatial differences in the diversity and community composition of the gut microbiota.

To determine the factors shaping the diversity and composition of the leaf and gut microbiota and to identify the core and transient components of the gut, it is necessary to analyze both environments simultaneously. Many metabarcoding studies have focused on a single factor^7,34–36^ and neglect possible multifactorial effects. Studies on the leaf microbiomes of multiple plant species are rare (see^12,13,37,38^ for exceptions) and the same applies to caterpillars^35,39,40^. These studies have focused on either the bacterial or fungal components. The fungal microbiome has been neglected, especially in caterpillars, although it may be richer than the bacterial microbiome^40^.

Using an extensive dataset from nine polyphagous caterpillar species sampled from five tree species at multiple sites in three geographically distant temperate forests, we aimed to (i) determine the factors shaping the composition and species richness of the bacterial and fungal microbiota of leaves and caterpillars, and (ii) compare the composition and richness of the leaf and gut microbiota to elucidate the origin/host fidelity of the gut microbiota. We hypothesized that in the case of the transient microbiota, the composition and richness of the gut microbiota of caterpillars feeding on different plant populations or species is different. If the community structure and richness were either affected by caterpillar species or constantly differed from the leaf microbiota, we expected the presence of specific gut microbiota.

## Results

### Dataset

Regarding the leaf samples, bacterial (μ = 9281 reads per sample; interquartile range (IQR) 2525–13,335) and fungal reads (μ = 4738; IQR 1702.5–6418) were represented by 10,965 and 4034 ASVs, respectively. On average, we classified 166.8 (SD ± 120.4) bacterial and 72.3 (SD ± 36.2) fungal ASVs per leaf sample. The bacterial ASVs occurred on average in 5.21 ± 0.142 samples (1.87 ± 0.051%), and the fungal ASVs occurred in 6.00 ± 0.260 samples (2.15 ± 0.093%).

The bacterial (μ = 7935 reads per sample; IQR 1571.5–9757) and fungal reads (μ = 5922; IQR 2028–7911) in the guts were represented by 12 004 and 9378 ASVs, respectively. On average, we classified 104.2 (SD ± 81.8) bacterial and 90.7 (SD ± 39.8) fungal ASVs per gut sample. The bacterial ASVs occurred on average in 7.44 ± 0.246 samples (0.84 ± 0.028%), and the fungal ASVs occurred in 9.54 ± 0.387 samples (1.08 ± 0.044%). The hierarchical taxonomic composition of the leaf and gut microbiota is shown in Figs. S1 (bacteria) and S2 (fungi).

### Factors shaping the leaf microbiota

The bacterial composition was shaped primarily by locality (explaining 10.94% of variability; df = 262, *F* = 19.36, *p* = 0.001), tree species (8.86%; df = 262, *F* = 6.27, *p* = 0.001) and their interactions (5.24%; df = 262, *F* = 2.65, *p* = 0.001) (Fig. S3), and irradiation (0.90%; df = 262, *F* = 3.18, *p* = 0.001). Fungal composition was shaped by the tree species (explaining 18.03% of variability; df = 264, *F* = 15.20, *p* = 0.001), locality (12.20%; *F* = 25.70, *p* = 0.001), and their interactions (7.12%; *F* = 4.29, *p* = 0.001) (Figs. S3, 1).

**Figure 1:**
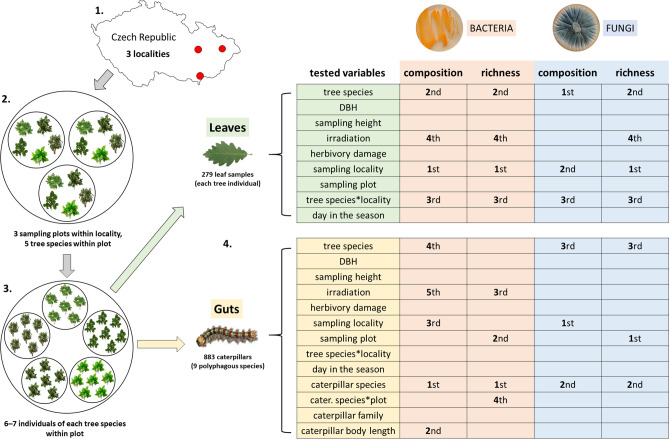
Sampling scheme for leaves and caterpillars with an overview of the effect of individual variables on the composition and richness of the associated bacteria and fungi. For significant variables, the order of their significance in the respective analyses is given based on Akaike’s information criterion (AIC), stepwise forward selection from permutational multivariate analysis of variance (PERMANOVA) (richness) and generalized linear models with Gamma distribution (composition). Only the significant interactions are shown.

**Figure 2:**
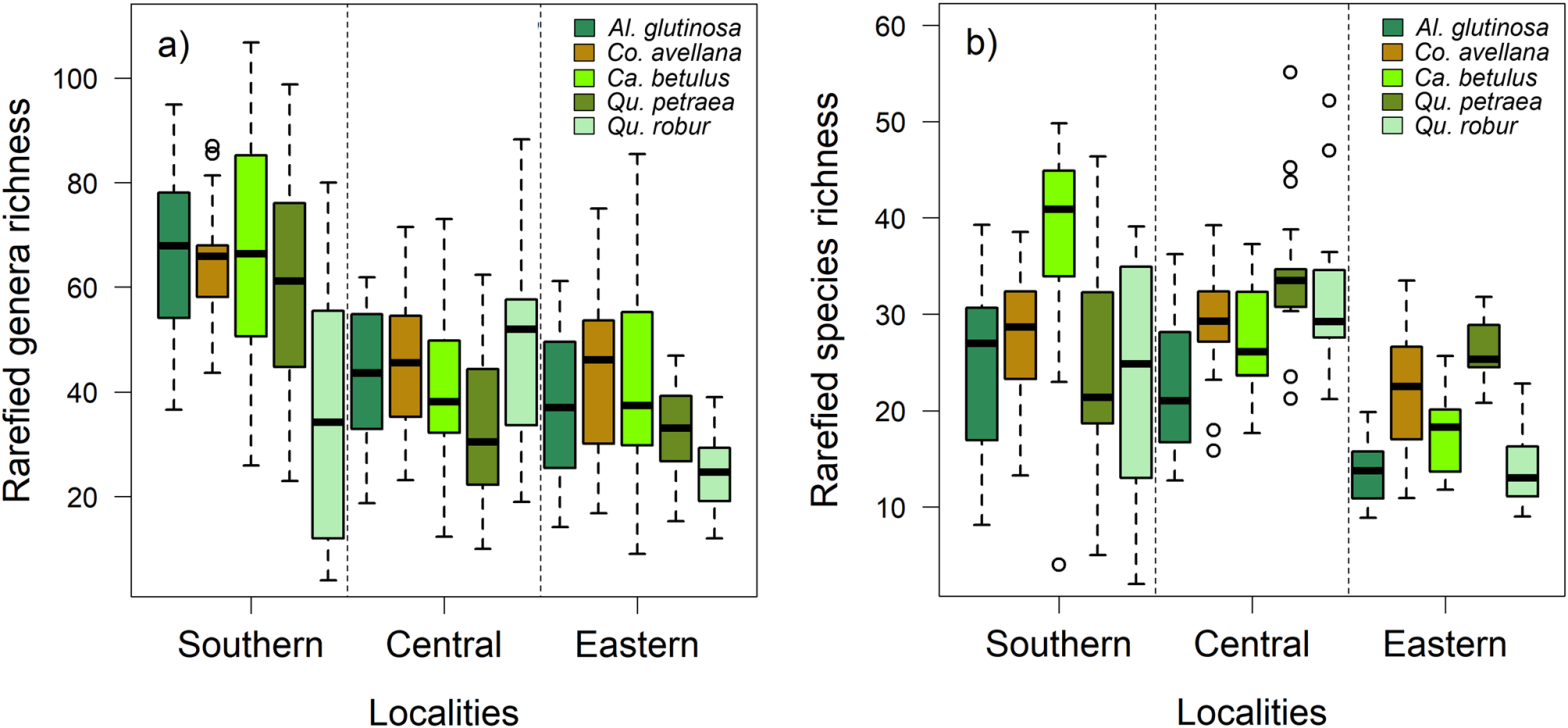
Rarefied richness of (**a**) bacterial genera and (**b**) fungal species at the tree species level within the sampling localities.

Bacterial richness mainly depended on the locality (df = 262, *F* = 40.22, *p* < 0.001), tree species (df = 262, *F* = 8.88, *p* < 0.001) and their interactions (df = 262, *F* = 4.74, *p* < 0.001) (Fig. 2a), and irradiation (df = 262, *F* = 5.02, *p* = 0.026). With increasing irradiation, the richness decreased (df = 262, *F* = 5.41, *p* = 0.021). Fungal richness mostly depended on the locality (df = 262, *F* = 48.54, *p* < 0.001), tree species (df = 262, *F* = 11.84, *p* < 0.001), and their interactions (df = 262, *F* = 8.58, *p* < 0.001) (Fig. 2b) and decreased significantly with increasing irradiation (df = 262, *F* = 5.45, *p* = 0.005) (Fig. 1).

### Factors shaping gut microbiota

**Figure 3:**
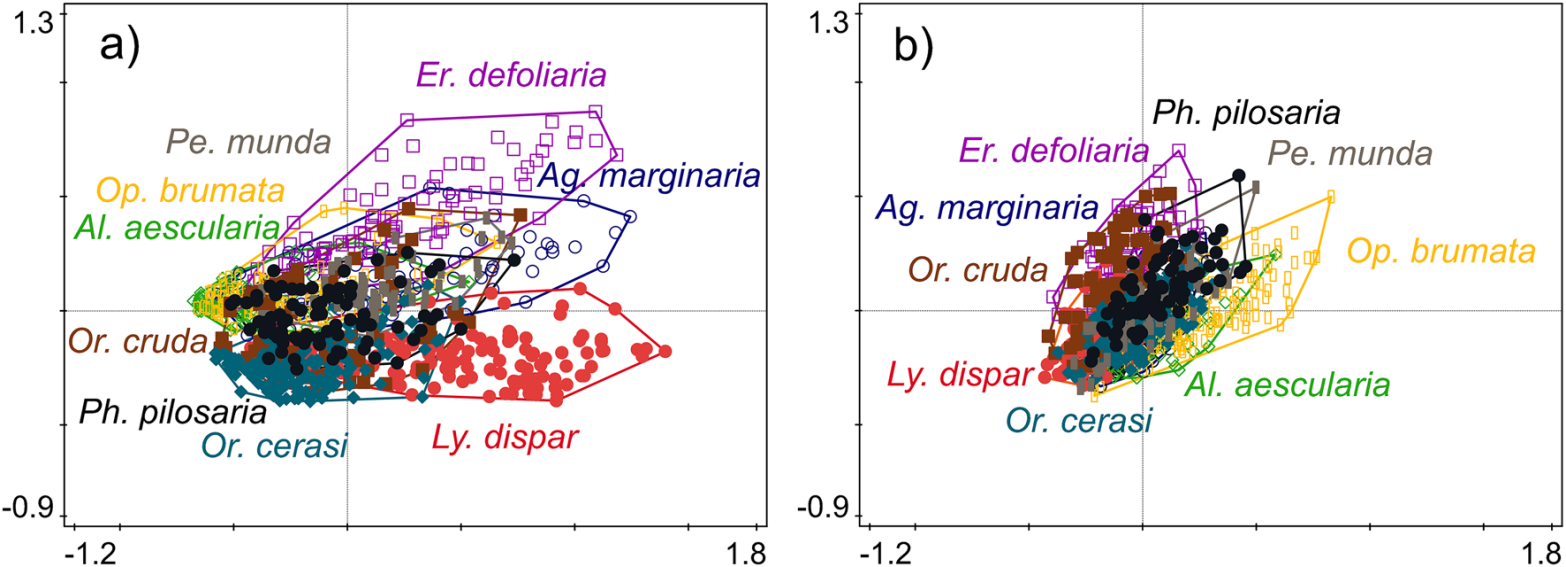
Principal coordinate analysis (PCoA) plots showing differences in (**a**) bacterial and (**b**) fungal microbiota composition among the guts of nine polyphagous caterpillar species.

The bacterial composition was primarily shaped by caterpillar species (explaining 21.10% of variability; df = 866, *F* = 30.36, *p* = 0.001; Figs. 3a, S4), then by the caterpillar body length (1.62%; df = 866, *F* = 18.62, *p* = 0.001), locality (0.94%; df = 866, *F* = 5.39, *p* = 0.001), tree species (0.91%; df = 866, *F* = 2.62, *p* = 0.001), and irradiation (0.20%; df = 866, *F* = 2.33, *p* = 0.011). The fungal composition was primarily shaped by the locality (explaining 8.40% of variability; df = 882, *F* = 5.16, *p* = 0.001) and the caterpillar species (4.02%; df = 882, *F* = 5.16, *p* = 0.001; Figs. 3b, S4), followed by the tree species (3.24%; df = 882, *F* = 6.65, *p* = 0.001) (Fig. 1).

**Figure 4:**
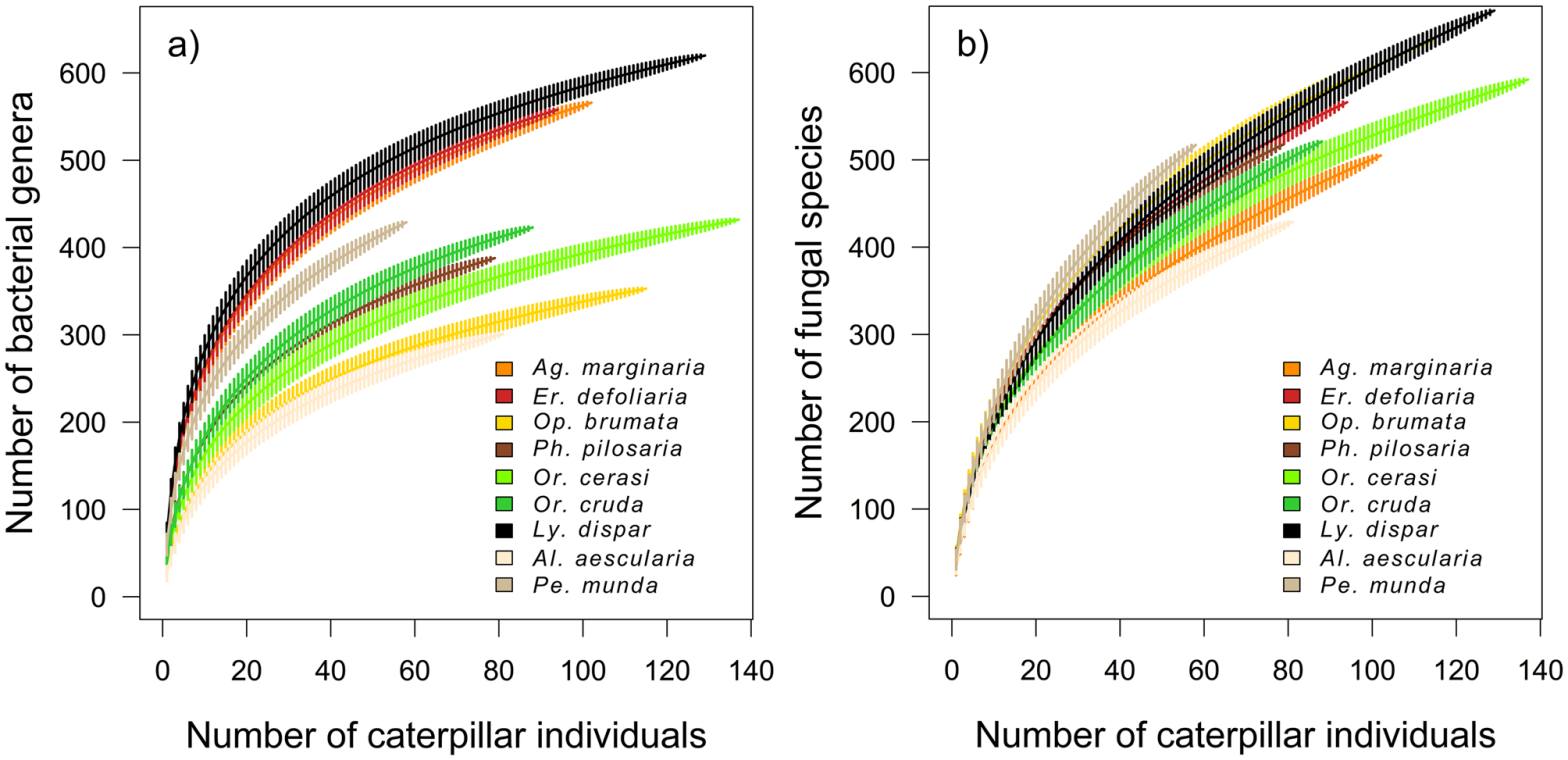
Accumulation curves (mean ± SD) of (**a**) bacterial genera and (**b**) fungal species associated with the guts of nine polyphagous caterpillar species.

The bacterial richness depended on the caterpillar species (df = 864, *F* = 62.47, *p* < 0.001; Fig. 4a), sampling plot (df = 864, *F* = 4.05, *p* < 0.001), and the irradiation (df = 864, *F* = 6.64, *p* = 0.001) with a peak bacterial richness at 60% of the irradiated crown. There was a significant interaction between caterpillar species and the sampling plot (df = 861, *F* = 3.49, *p* < 0.001). Fungal richness depended on the sampling plot (df = 861, *F* = 18.06, *p* < 0.001), followed by the caterpillar species (df = 861, *F* = 10.32, *p* < 0.001; Fig. 4b) and the tree species (df = 861, *F* = 5.52, *p* < 0.001) (Fig. 1).

### Comparison of the leaf and gut microbiota

**Figure 5:**
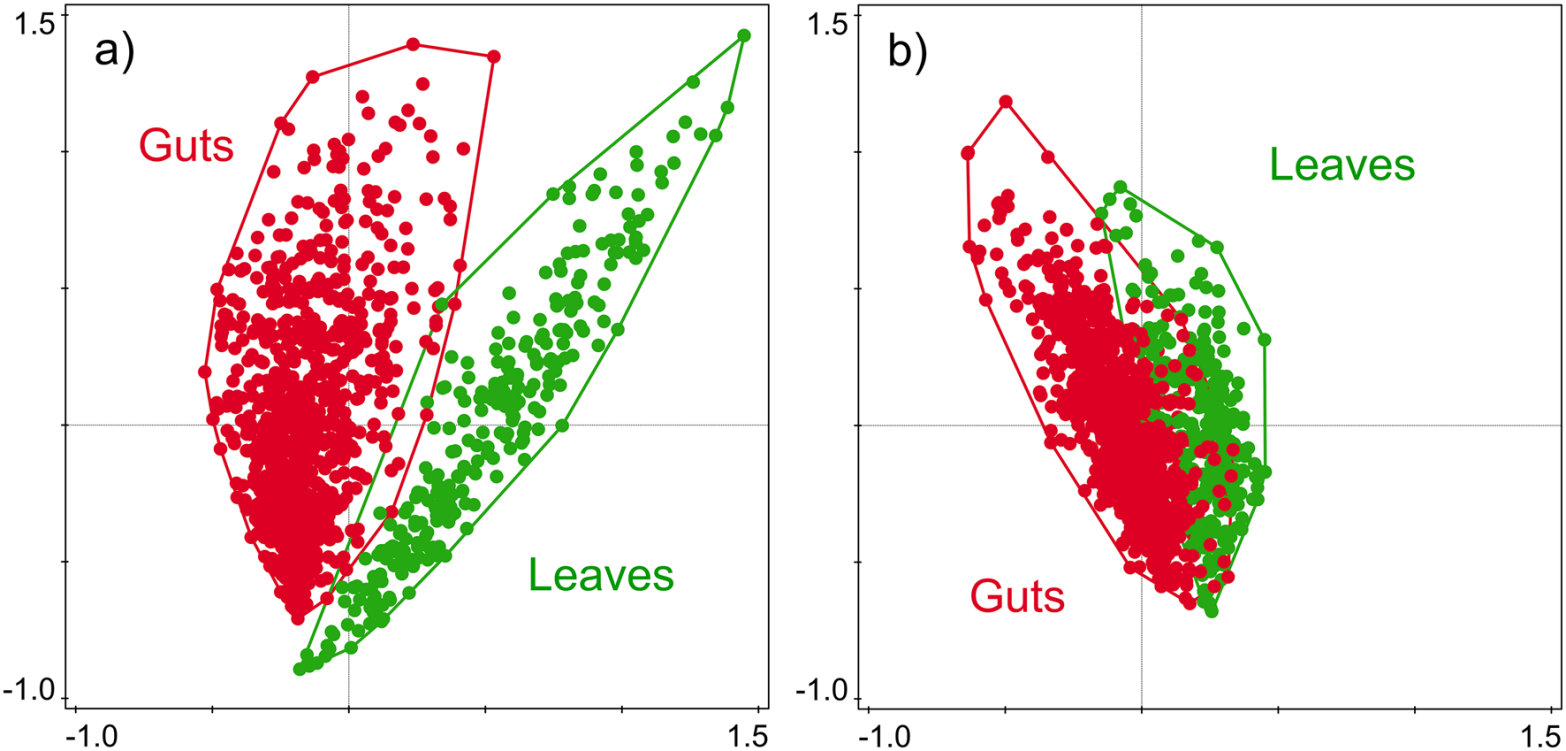
Similarity comparison of (**a**) bacterial and (**b**) fungal microbiota composition between caterpillar guts and host tree leaves.

The composition of the bacterial microbiota of leaves and guts significantly differed, explaining 7.40% of the variability (df = 1160, *F* = 92.66, *p* = 0.001; Fig. 5a). The composition of fungal microbiota also differed, explaining 1.14% of the variability (df = 1161, *F* = 13.43, *p* = 0.001; Fig. 5b). The dispersion in β-diversity of samples was greater for guts than for leaves in the bacteria (df = 1159, *F* = 16.90, *p* = 0.001) but not in the fungi (df = 1160, *F* = 3.06, *p* = 0.087; Fig. S5). The quantitative similarity between the leaves and guts (i.e., leaf–gut similarity) was higher for fungal microbiota than for the bacterial microbiota (V = 8091, *p* < 0.001; Figs. 5a,b and S6). The leaf–gut similarity did not differ among the host plants (df = 877, χ^2^ = 1.45, *p* = 0.835 and df = 878, χ^2^ = 8.97, *p* = 0.062 for bacteria and fungi, respectively) or among caterpillar species for the fungal microbiome (df = 874, χ^2^ = 9.15, *p* = 0.329) but differed among caterpillar species for the bacterial microbiome (df = 873, χ^2^ = 19.70, *p* = 0.012) and was significantly lower in *A. aescularia* and *O. brumata* (df = 873, z = − 2.68, *p* = 0.007 and df = 873, z = − 2.01, *p* = 0.045, respectively).

**Figure 6:**
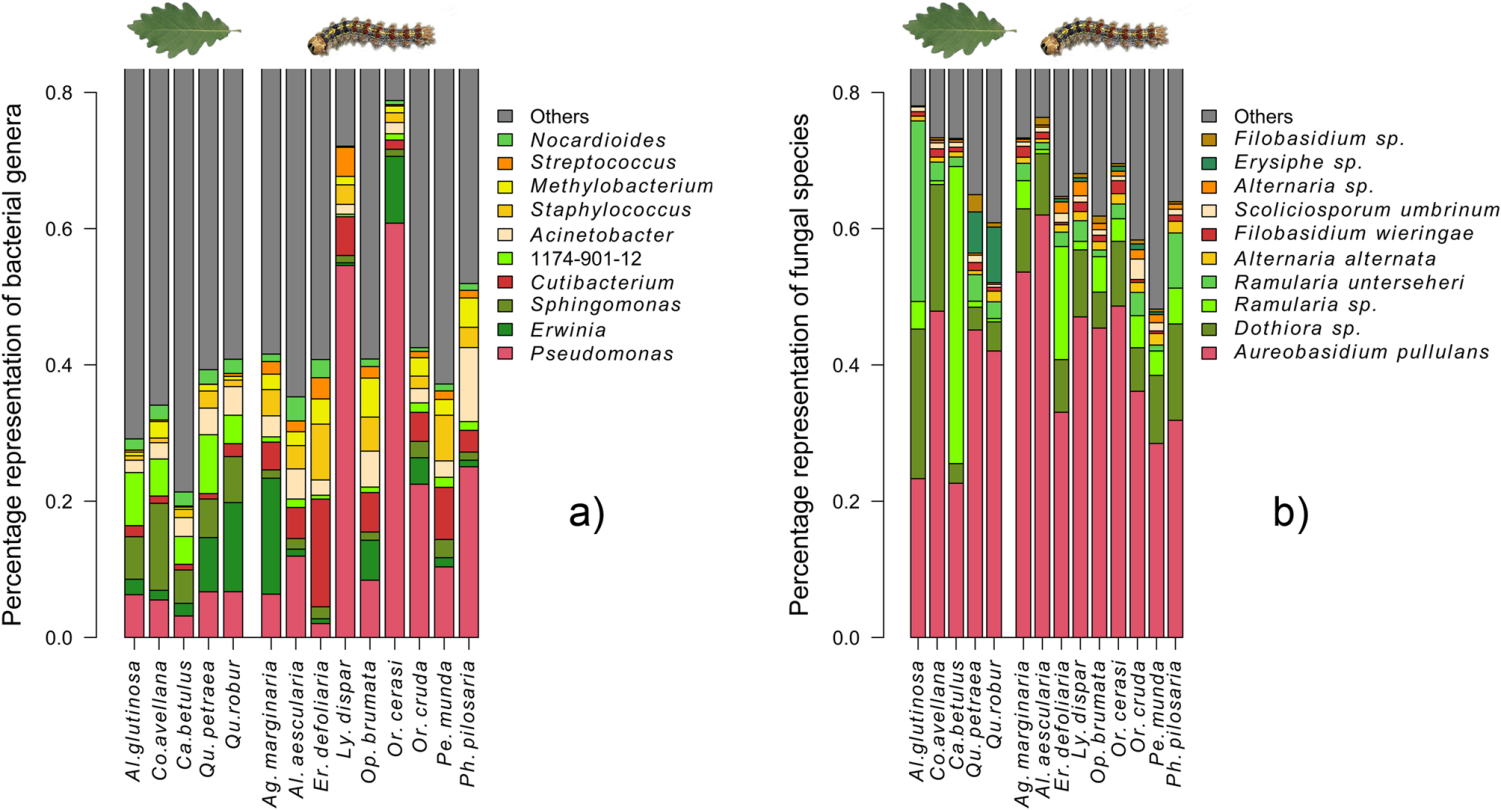
Composition of (**a**) bacterial and (**b**) fungal microbiota associated with caterpillar guts and host tree leaves. The 10 most abundant bacterial genera and fungal species are shown.

Among the 10 most abundant bacteria and fungi, *Streptococcus* (bacteria) was significantly associated with the guts (*p* < 0.05), whereas *Sphingomonas* (bacteria) and *Erysiphe* (fungus) were significantly associated with leaves (*p* < 0.05) (Fig. 6a,b). All the indicator taxa are shown in Table S1.

Rarefied bacterial genera richness was higher in the leaves than in the guts (χ^2^ = 9.82, *p* = 0.007; 47.3 [SD ± 20.2] and 40.3 [SD ± 21.7] genera per sample in the leaves and guts, respectively), whereas the rarefied fungal species richness was higher in the guts than in the leaves (χ^2^ = 15.91, *p* < 0.001; 30.0 [SD ± 9.2] and 25.9 [SD ± 9.3] species per sample in the guts and leaves, respectively). In the leaves and guts, the bacterial genera richness was higher than the fungal species richness (V = 36 614, *p* < 0.001; V = 279 457, *p* < 0.001) (Fig. S7).

## Discussion

Leaves harbor diverse and abundant bacterial^12,38^ and fungal^13,41^ microbial communities. However, the high overall richness in the caterpillar guts contradicts previous findings on species-poor bacterial communities^7,30,39^ Contrary to the findings of our previous study^40^, the bacterial microbiome was richer than the fungal microbiome, especially considering that bacterial richness was estimated at the genus level and the fungal richness was estimated at the species level. On the other hand, our previous study was aimed primarily at fungal microbiomes of leaves and caterpillar guts, and bacteria were studied only marginally. Therefore, the number of bacterial samples in that study was low. Moreover, in our previous study, we focused mainly on the surficial microbiota (epiphytes) and used different primer sets and bioinformatic tools. In that study, we worked with OTUs, bacteria were sequenced in 300 bp paired-end mode and identified using the GreenGenes database, and fungi were identified only to the genus level, altogether resulting in discrepancy between both studies. Nevertheless, the quantification of the 16S and ITS2 DNA in the initial samples is necessary to assess the proportional representation of bacteria and fungi in the gut microbiota. Moreover, the extent to which microbial communities obtained by metabarcoding are composed of legacy DNA from dead or dormant cells and spores remains to be determined^42^.

The bacterial and fungal components of the leaves were significantly affected by tree species, which is a typical pattern^43^. However, they significantly interacted with locality, implying that trees at different localities harbored specific microbial assemblages. This is in contrast with the findings of a recent study that suggested low variability in the leaf microbial diversity among individual sites^44^. However, these findings are aligned with the leaf microbes being acquired from the environment, but their survival is filtered by the plant^45^. The microbiomes of a focal host species may be affected by the local plant community composition and diversity^31,37,44^. These measurements were beyond the scope of our study but may be responsible for the spatial differences recorded.

Irradiation significantly affected the fungal richness and bacterial composition and species richness. Sun exposure is a good predictor of fungal abundance^46^ and diversity^47^. For the bacteria, Stone and Jackson^48^ found distinct community composition across canopy positions, with speculative attributability to radiation. However, shaded leaves are less exposed to rain, which changes the bacterial composition. Moisture availability may have had a substantial impact on the leaf microbiota^49^. Desiccation, especially in combination with UV radiation, strongly limits microbial populations^50^. However, in the leaf bacteria, UV radiation has been found to have no effect on the species richness^51^.

In caterpillars, bacterial composition and richness were primarily shaped by the caterpillar species. In fungi, the role of the host species is secondary, with these being primarily shaped by spatial variables. Strong interspecific differences were found in the current study, which is in contrast with the findings of numerous studies that similar communities are shared among caterpillar species. This implies a relatively low level of importance for the host physiological environment in structuring microbial communities and emphasizing the dietary effect^19,29,35,39^. In contrast, independent of diet, there are interspecific differences in the physiochemical conditions of the gut, which exert strong selection pressure on microbiota^5,17^. In this context, the associated differences in the gut microbiota were in line with other findings from the literature.

Bacterial composition was significantly affected by the caterpillar body length. Changes in the community composition are attributed to the increasing importance of gut filtering throughout the caterpillar life cycle^7,28,52^. As caterpillars grow, less oxygen penetrates the gut lumen, which promotes the development of facultative anaerobic bacteria dominated by Enterobacteriaceae, which decreases the diversity^23^. This was not reflected here because we only sampled similarly sized caterpillars (3–4th instar) to suppress the effects of the developmental stage. This protocol did not result in a dataset composed of same-instar caterpillars, and some effect from body length may be attributable to this effect.

Apart from the strong effect of the caterpillar species, differences in the composition and richness of the gut bacterial microbiome were shaped by irradiation, spatial variables, and their interaction with the caterpillar species. The climatic and ecological factors of host habitats are known to affect the insect gut bacterial composition^53,54^. However, given that the conditions of the individual plots affect individual caterpillar species differently, the effect of the environmental conditions remains unresolved. The bacterial richness and composition may be strongly affected by biotic conditions, especially parasitoid infection^26^. The spatial differences in the parasitism rate and the parasitoid community composition are well known^55,56^. Although we tried to eliminate all the parasitized samples prior to processing, the parasitoid juvenile stages may have been overlooked and could have contributed to the differences in the bacterial composition and richness.

Host tree species also significantly affected the gut fungal and bacterial microbiomes. Bacterial gut communities are strongly influenced by diet^57^. Here, however, the leaf bacteria were primarily affected by the spatial variables. Therefore, the bacterial gut content may not be affected by the microbial composition of the diet, but rather by its quality including the protein and carbohydrate content^20^, and plant secondary metabolites^25^. This places strong selective pressure on the gut microbiota. This pressure seems to be less important for fungal components, which are primarily affected by the spatial variables either in the diet or the gut.

The composition of the bacterial and fungal gut components significantly differed from those of the leaves. However, this difference was much more pronounced in the bacteria. Caterpillars move over relatively long distances when feeding^58^, and *Orthosia* spp. are occasional entomophages^59^. They likely sampled a much larger microbial pool than that reflected in the relevant leaf sample, which may have contributed to their low level of similarity. The bacterial richness was higher in the leaves than in the guts, whereas fungal components showed an opposite pattern, suggesting that the fungi may be less filtered than the bacteria. The composition of the leaf bacteria was balanced, whereas in the guts there was a higher level of host-interspecific variability. In contrast, the fungal component of both leaves and guts was dominated by *Aureobasidium pullulans*, *Ramularia*, and *Dothiora*, highlighting an occurrence of environmental acquisition greater than in bacteria.

This finding was corroborated by analysis of the factors that form individual microbial components of the gut. In fungi, the caterpillar species was a secondary factor explaining less variability than the spatial variables including the locality and the sampling plot, which predominantly shaped the richness and composition of the diet. However, the bacterial component of the guts was shaped primarily by the caterpillar species and was affected by the caterpillar body length, indicating a greater involvement of gut filtering. The bacteria in the diet varied considerably among individual localities. However, this was not reflected in the gut bacteria. The spatial variability of bacteria manifested at the level of individual localities, which may reflect the host’s adaptation to local conditions. These results indicate that the fungal component of the gut is more transient, whereas bacteria form a core component.

The leaf–gut similarity of the bacterial—but not the fungal—components differed significantly among caterpillar species, suggesting that some species have lower leaf–gut similarities (specifically *A. aescularia* and *O. brumata*). This suggests a higher involvement of resident (core) bacterial taxa or stronger environmental filtering. This may be explained by their similar life histories and dispersal strategies. Unlike the remainder of the study species, adults remain active during winter, occurring in high abundances^60^. For both species, ballooning dispersal, in which caterpillars use silk to move through the air, was documented^61^. Both strategies could contribute to sampling different microbial pools from the environment. Extreme winter conditions may alter the adult gut microbiota, which may be vertically transferred to the offspring through contamination of the egg surface^23,28^. While the bacterial microbiota of some hosts (*P. munda*, *A. aescularia*, *P. pilosaria*, *O. brumata*) were relatively similar, other species (*E. defoliaria*, *L. dispar*) hosted more specific bacterial consortia, suggesting diversity in the direction of environmental filtering.

Our study highlights the complexity of the factors shaping leaf and caterpillar gut microbiota, which makes it difficult to draw conclusions, even with a large dataset. Other important factors were not accounted for in this study, namely the interactions within and among microbial groups that are abundant and of considerable importance in the leaves^62,63^ and the gut^3,64^. The relatively static physiochemistry of the host as well as the dynamic microbe-microbe, microbe-host and host-mediated microbe-microbe interactions are likely the drivers of microbiota community composition^43^, as applied in both environments. Future studies using a community-level approach may clarify the relative importance of stochastic and deterministic processes in governing the gut microbiota assembly and how this importance varies through space and time. A functional approach using transcriptomics, which identifies biologically active taxa, would complement these studies, and elucidate the link between the core component of the gut and its significance for the host.

## Materials and methods

### Sampling of leaves and caterpillars

Field sampling was conducted at the end of April to mid-May, 2018 at three remote localities in the temperate floodplain forests of Moravia, Czech Republic: southern (48.8926N, 17.0700E; 170 m a.s.l.), central (49.6932N, 17.1399E; 225 m a.s.l.), and eastern (49.7918N, 18.2061E; 220 m a.s.l.) (Fig. S8). At each locality, we sampled the leaves of five tree (Fagales: *Quercus robur*, *Q. petraea*, *Corylus avellana*, *Carpinus betulus*, and *Alnus glutinosa*) and nine caterpillar species (polyphagous leaf-chewers, Lepidoptera—Noctuidae: *Orthosia cerasi*, *O. cruda*, and *Perigrapha munda*; Geometridae: *Agriopis marginaria*, *Alsophila aescularia*, *Erannis defoliaria*, *Operophtera brumata*, and *Phigalia pilosaria*; and Erebidae: *Lymantria dispar*). Within each locality, we set three remote sites (sampling plots) containing all the tree species, each represented by six to seven individuals (Fig. 1). The caterpillars were sampled manually or by using 1-m^2^ beating sheets. Each individual was captured using sterilized tweezers, transferred to a 1.5-ml centrifuge tube with 98% ethanol, and the post-mortem length was then measured. To minimize the combined effect of the developmental stage and the locality (localities were sampled consecutively) on the composition and diversity of the microbiota^7^, we sampled 3–4th instars of the given species in each sampling plot.

Simultaneously, we sampled the host tree leaves. Within each tree, we randomly selected five leaves, cut the middle parts (2 cm^2^ segments; i.e., 10 cm^2^ per sample) using sterilized tweezers and scissors, and transferred them to a 1.5-ml centrifuge tube with 98% ethanol. Given that herbivory is known to generate variations in the within-host microbial fitness and alter the structure of the leaf microbiota^65,66^, we assessed the effect of herbivory by selecting approximately half of the samples (n = 136) from leaves with herbivory damage and half (n = 143) from pristine leaves. For each tree from which we had leaf and caterpillar samples, we estimated (based on three collectors) the irradiated proportion of the crown, measured the sampling height above the ground using a digital laser distance meter (HECHT^®^ 2006; Hecht Motors Inc., Prague, Czech Republic), and measured the diameter at breast height. The tubes with the caterpillar and leaf samples were stored at − 32 °C. To maintain a balanced sampling design, leaf samples from 279 tree individuals and 883 caterpillars with the best overlap among tree individuals, tree species, sampling plots, and localities were selected for further processing (Table S2).

### Identification of caterpillars

The caterpillars were identified at the morphospecies level using standard identification keys, field guides, and online databases (Table S3). Specimens that could not be reliably assigned to a morphospecies (e.g., congeneric species; 231 individuals) were subjected to the DNA barcoding of cytochrome oxidase subunit I (COI) following Hrcek et al.^67^. We used DNA extracted from the guts (see below). PCR products were sequenced in the forward or reverse direction using an ABI 3730XL sequencer (Macrogen Europe, Amsterdam, Netherlands). Specimen records with sequences are accessible on BOLD (dataset DS-SYMB; DOI 10.5883/DS-SYMB).

### Processing of caterpillars and leaves

Each caterpillar was washed through vortexing in a 1.5-ml tube with 98% ethanol at 2100 rpm for 90 s, transferred to a clean 1.5-ml tube, and washed in a 1-ml sterile solution of 1% Tween 80 and phosphate-buffered saline (PBS) (Sigma-Aldrich, Saint Louis, MO, USA) at 2100 rpm for 45 s to minimize contamination by surficial microbiota. The gut content was separated using a sterilized scalpel, needle, and minute pins onto paraffin wax sterilized with flamed ethanol^68^ and transferred to a new 1.5-ml tube with 100 μl of 1× PBS. The leaf samples in the 1.5-ml tubes with 98% ethanol were vortexed at 2100 rpm for 45 s and then centrifuged at 5400×*g* for 15 min at 4 °C. The supernatant was discarded, and the residual ethanol was evaporated at 55 °C for 45 min. Subsequently, leaf samples (tissue together with the DNA pellet from their surface) were resuspended in 200 μl of 1× PBS solution and stored at − 32 °C for subsequent DNA isolation.

### DNA metabarcoding of bacteria and fungi

The DNA was extracted at the level of individuals from the guts (n = 883) and leaves (n = 279) using a NucleoSpin Tissue DNA Isolation Kit (Macherey–Nagel, Düren, Germany) following the manufacturer’s protocol. The samples were repeatedly crushed in 1.5-ml tubes using plastic pestles and liquid nitrogen before cell lysis. In samples with higher amounts of the input tissue, we adequately increased the volume of enzymes and buffers used for (pre)lysis and subsequent DNA binding steps. To ensure broad bacterial and fungal diversity recovery, we used highly degenerate primers, which can significantly reduce the recovery of plant-originating sequences (chloroplasts). For the amplification of the fungal ITS2 rRNA region, we used ITS3_KYO2 5ʹ-GATGAAGAACGYAGYRAA-3ʹ (forward) and ITS4_KYO3 5ʹ-CTBTTVCCKCTTCACTCG-3ʹ (reverse)^69^, and for the bacterial V5–V6 16S rRNA region, we used 799F 5ʹ-CMGGATTAGATACCCKGG-3ʹ (forward) and 1115R 5ʹ-AGGGTTGCGCTCGTTG-3ʹ (reverse)^70,71^ with barcodes added to the 5ʹ end of both primers, enabling the identification of each sample. All the PCRs were performed in triplicate to minimize the effects of stochastic amplification. The amplification of the ITS2 rRNA gene region was performed as described by Toju et al.^69^ with minor modifications consisting of initial denaturation at 95 °C for 3 min; 35 cycles at 94 °C for 30 s, 55 °C for 60 s, 72 °C for 60 s; and a final extension at 72 °C for 10 min. The amplification of the 16S rRNA gene region consisted of initial denaturation at 94 °C for 4 min; 35 cycles at 94 °C for 45 s, 50 °C for 60 s, 72 °C for 75 s, and a final extension at 72 °C for 10 min. Each PCR reaction (25 μl) consisted of 9.4 μl molecular biology grade water (New England BioLabs, Ipswich, MA, USA), 0.5 U KAPA2G Robust HotStart DNA Polymerase, 5 μl of 5× KAPA2G Buffer B, 5 μl of 5× KAPA2G Enhancer (all Kapa Biosystems, Wilmington, NC, USA), 0.5 μl of 10 mM dNTP Mix (Thermo Fisher Scientific, Waltham, MA, USA), 0.8 μM of each primer, and 2 μl of genomic DNA. All the PCR products were analyzed using 1.5% agarose gel. We pooled triplicate PCR reactions of individual samples within each “plate library” (96 samples). The amplicons of the specific length from individual libraries were excised from the 2% agarose gel and purified using a QIAquick Gel Extraction Kit (Qiagen, Hilden, Germany). The DNA concentration was measured using a Qubit dsDNA BR Assay Kit (Thermo Fisher Scientific), and we equalized concentrations within all the libraries to 20 ng/μl. Individual “plate libraries” were subjected to DNA ligation of sequencing adapters and library-unique multiplex identifiers using the KAPA Hyper Prep Kit, and were subsequently quantified using a KAPA Library Quantification Kit (both Kapa Biosystems). The equimolar proportions of the individual “plate libraries” were pooled, creating one final library of fungal samples and a second of bacterial samples at 7.5 ng/μl. The fungal library was subjected to paired-end sequencing on a MiSeq instrument, producing 2 × 300 bp reads (four runs in total), whereas the bacterial library was subjected to single-end sequencing on the NextSeq 500 (Illumina Inc., San Diego, CA, USA) (one run), producing a 1 × 150 bp read at the Genomics Core Facility, CEITEC (Masaryk University, Brno, Czech Republic). In this study, bacterial sequences represented 39.0% and fungal sequences represented 39.8% of the total NextSeq and MiSeq sequencing outputs. The remaining sequences were dedicated to another study. The raw demultiplexed sequencing data with sample annotations are available at the NCBI Bioproject website (https://www.ncbi.nlm.nih.gov/bioproject/) under the accession number PRJNA694554.

### DNA metabarcoding data processing

The sequencing data were processed using QIIME 2.0 2020.2^72^. The raw reads were demultiplexed and quality filtered using the q2‐demux plugin, and in the case of the fungal datasets, the ITS region was extracted using the q2-ITSxpress plugin^73^. Afterwards, the reads were denoised using the DADA2 algorithm^74^ and a feature table with counts of amplicon sequence variants (ASVs) per sample was produced. The taxonomy was assigned using the q2‐feature‐classifier classify-sklearn^75^ using a trained naïve Bayes classifier against the SILVA_138_SSURef_Nr99 bacterial reference database^76^ and UNITE QIIME release for Fungi version 8.0^77,78^. We obtained an ASV table with 27,552,665 bacterial and 6,679,221 fungal reads. Further, we identified contaminant ASVs using the “decontam” package^79^ based on the prevalence method with extraction controls as negatives (three per each 96-well plate). The probability threshold below which the null hypothesis of non-contamination was rejected was 0.1. We discarded 353 bacterial and 291 fungal ASVs (2.92% of reads; Table S4) and removed unassigned reads (3.04%) and those associated with chloroplasts and mitochondria (46.9%). Finally, 9,586,289 bacterial (7,006,293 gut; 2,579,996 leaf) and 6,551,306 fungal reads (5,229,511 gut; 1,321,795 leaf) were used for analysis.

### Statistical analyses

The data were analyzed using R 4.0.2^80^ and Canoco 5.0^81^. For hierarchical visualization of the recovered fungal and bacterial composition of the leaf and gut microbiota, we used Krona charts^82^. The bacterial ASVs were analyzed at the genus level (only a small number of ASVs could be classified to the species level), whereas the fungal ASVs were analyzed at the species level. For the bacterial and fungal taxa, the number of reads, and the variables entering the analyzes, see Table S5. To compare the bacterial genera/fungal species richness, the number of reads in each sample was rarefied to 400. Rickettsiales (i.e., *Rickettsia* and *Wolbachia*) were excluded from the analyses of gut composition as they are intracellular parasites, likely originating from gut cells instead of the lumen. However, they were not excluded from the leaf analyses, in which they commonly survive^83^. However, this group was excluded from both datasets for comparison of the leaf and gut composition. For the final generalized linear models, we checked the possible collinearity of variables using the generalized variance inflation factor from the “car” package^84^ adjusted to the given degrees of freedom, potentially excluding variables exceeding the threshold of > 2.

### Ethics statement

No specific permissions were required to collect insect and plant specimens, because the study species do not include any species at the risk of extinction, according to the IUCN Policy Statement on Research Involving Species at Risk of Extinction, or endangered species of wild fauna and flora according to the Convention on the Trade in Endangered Species of Wild Fauna and Flora. Voucher specimens for all the plant and insect species described in the manuscript are deposited in the collection of the University of Ostrava (with deposition numbers from DS_SYMB_F001 to DS_SYMB_F279 for plants, and from DS_SYMB_C001 to DS_SYMB_C883 for insects).

### Factors shaping the leaf and gut microbiota

To determine the most important factors shaping the composition of the leaf and gut microbiota, we performed permutational multivariate analysis of variance (PERMANOVA) using the “vegan” package^85^ with 999 permutations and distance matrices calculated using the Bray–Curtis method separately for the bacterial and fungal datasets. For the leaf microbiota, we used two groups of explanatory variables, characterizing the host tree (species, diameter at breast height, sampling height, irradiation of crown, and herbivory damage) and the environment (locality, sampling plot, and day in the season). For the gut microbiota, we added a third group of variables characterizing the host caterpillar (species, family, and body length). The final models were built using stepwise forward selection based on Akaike’s information criterion (AIC). The resulting models were accompanied by principal coordinate analyses (PCoA) of both datasets tested by Monte Carlo permutation tests for the significance of correlation with 999 permutations. The caterpillar species was used as an explanatory variable, and for the fungal dataset, locality was used as a covariate, which explained more variability than the caterpillar species in PERMANOVA.

We analyzed the rarefied richness using generalized linear models with Gamma distribution, except for a linear model for the gut microbiota fungal dataset, which were built using stepwise forward selection based on AIC from the set of explanatory variables characterizing the trees, environment, and caterpillars for the gut microbiota only. For the gut microbiota, we compared the distribution of the rarefied richness for the bacterial and fungal datasets using the paired Wilcoxon signed-rank test.

### Comparison of the leaf and gut microbiota

We used PERMANOVA to compare the bacterial/fungal composition between the leaves and the guts. Given that multivariate variation among the test groups may, in the case of an unbalanced number of samples, compromise the PERMANOVA results, we added PERMDISP2 procedure for the analysis of multivariate homogeneity of group dispersions (variances) based on the Bray–Curtis distance, measuring the distance to the group centroids^86^. The models were accompanied by PCoA for both datasets, with each tested using the Monte Carlo permutation test for the significance of correlation with 999 permutations. The analyses were supplied by bar plots depicting the 10 most abundant bacterial genera and fungal species. We calculated the quantitative similarity between each gut and its host tree leaf sample using the Renkonen index^87^. We compared the distribution of the leaf–gut similarity for the bacterial and fungal datasets using paired Wilcoxon signed-rank tests. We analyzed which explanatory variables affected the similarity using generalized linear models with binomial distribution built by stepwise forward selection. For the final model, the contrasts were set to sum, which compares the mean of a dependent variable for a given level to the overall mean of the dependent variable.

The rarefied bacterial genera/fungal species richness of the gut and leaf microbiota was compared using generalized linear mixed models with Gamma distribution and the sampling plot as random terms using the “lme4” package^88^. The significance of the models was determined by comparing them with relevant null models. We identified the indicator bacterial genera/fungal species for the leaves and guts and separately for the gut microbiota of each caterpillar species and the leaf microbiota of each tree species using the IndVal method from the “labdsv”^89^ and “indicspecies”^90^ packages, which generates a value indicating the frequency and relative abundance of reads^91^ and by using multi-level pattern analysis^89,90^ with adjusted *p*-values to correct for multiple comparisons using Benjamini–Hochberg corrections^92^.

## Supplementary Information

Supplementary Figure S1.

Supplementary Figure S2.

Supplementary Information.

Supplementary Table S1.

Supplementary Table S4.

Supplementary Table S5.

## Data Availability

Caterpillar specimen records with sequences are accessible on BOLD (dataset DS-SYMB; 10.5883/DS-SYMB). Bacterial and fungal raw demultiplexed sequencing data with sample annotations are available at the NCBI Bioproject website (https://www.ncbi.nlm.nih.gov/bioproject/) under the accession number PRJNA694554. An overview of the bacterial and fungal taxa, the number of reads, and the variables entering the analyzes is included in the Supplementary Information (Table S5).
