# Supplementary material for: Fungi are more transient than bacteria in caterpillar gut microbiomes
Source: Sci Rep. 2022 Sep 16;12:15552. doi: 10.1038/s41598-022-19855-5 (PMC9481635; doi:10.1038/s41598-022-19855-5)
Supplement: Supplementary file 1 — Supplementary Figure S1. [file 41598_2022_19855_MOESM1_ESM.html]

Javascript must be enabled to view this page.

magnitude

guts
leaves

 1861719
 1246213

 1861719
 1246213

 216
 459

 216
 459

 216
 459

 216
 459

 216
 459

 4921
 22906

 3860
 16316

 334
 237

 334
 237

 17
 28

 317
 209

 3189
 15201

 3123
 15054

 7
 140

 58
 318

 878
 3129

 10
 221

 1462
 5547

 29
 90

 70
 148

 609
 5461

 5
 30

 5
 30

 26
 33

 26
 33

 2

 2

 2

 216
 579

 216
 579

 216
 579

 22
 20

 22
 20

 22
 20

 53
 223

 53
 223

 53
 223

 32
 47

 32
 47

 32
 47

 12

 12

 12

 0
 9

 0
 9

 0
 9

 394
 1189

 326
 1062

 326
 1062

 16
 90

 97
 135

 15
 75

 35
 324

 105
 338

 0
 23

 32
 68

 32
 68

 32
 68

 36
 26

 36
 26

 36
 26

 0
 21

 0
 21

 0
 21

 0
 12

 0
 12

 0
 12

 50
 248

 11

 11

 11

 39
 248

 39
 248

 39
 248

 5
 11

 5
 11

 5
 11

 5
 11

 2
 2

 2
 2

 2
 2

 2
 2

 50
 162

 50
 162

 50
 162

 50
 162

 76
 523

 76
 523

 76
 523

 76
 523

 475
 4407

 22
 188

 22
 188

 22
 188

 453
 4219

 236
 2632

 41
 230

 195
 2320

 31
 186

 31
 186

 9
 26

 9
 26

 9
 26

 9
 26

 0
 11

 0
 11

 0
 11

 0
 11

 0
 4

 0
 4

 0
 4

 0
 4

 0
 7

 0
 7

 0
 7

 0
 7

 357410
 183658

 4177
 11427

 55
 378

 0
 10

 0
 10

 239
 509

 239
 509

 239
 509

 2764
 8137

 420
 877

 420
 877

 1619
 5378

 319
 787

 340
 1580

 79
 298

 134
 510

 54
 103

 0
 25

 0
 21

 576
 1290

 576
 1290

 921
 2254

 921
 2254

 921
 2254

 343049
 145409

 16
 11

 16
 11

 16
 11

 2355
 166

 2355
 166

 1998
 159

 30

 35

 28
 7

 167

 86

 2681
 4431

 2681
 4431

 2

 1339
 4421

 1271
 10

 69

 16
 9

 16
 9

 16
 9

 47210
 7410

 30820
 3648

 147

 29416
 3470

 1257
 162

 0
 16

 770
 219

 770
 219

 9260
 2529

 9260
 2529

 6323
 860

 2010
 86

 2176
 427

 979
 139

 1072
 36

 86
 172

 12
 154

 12
 154

 7

 7

 18

 18

 10
 6

 10
 6

 0
 4

 15055
 12480

 502
 1794

 502
 1794

 25
 33

 25
 33

 4721
 2260

 4721
 2260

 283
 284

 283
 284

 4287
 2039

 5

 684
 793

 381
 419

 2759
 438

 165
 302

 293
 87

 3740
 4554

 3740
 4554

 68
 41

 22

 4
 11

 16

 22

 15

 15

 15

 11397
 2869

 11397
 2869

 176
 98

 8874
 1584

 759
 519

 39
 11

 304
 2

 1212
 585

 101604
 43957

 179
 40

 179
 40

 6
 39

 6
 39

 3449
 118

 3449
 103

 0
 15

 1569
 2045

 301
 537

 3

 1265
 1508

 61
 25

 8
 20

 41
 5

 1477
 285

 1329
 285

 148

 2795
 1713

 2723
 1476

 13

 35
 20

 11
 217

 13

 52
 17

 0
 17

 5774
 7054

 170
 247

 28
 14

 3053
 4642

 6

 775
 53

 789
 777

 6

 40
 85

 5

 241
 169

 90

 202
 238

 57
 73

 47983
 26967

 283
 96

 151
 112

 5618
 4223

 467
 136

 29
 18

 10405
 1272

 11

 12
 249

 9

 59
 30

 11

 1549
 1233

 204
 34

 227

 818
 75

 204
 20

 27926
 19453

 0
 11

 0
 5

 31465
 3782

 1094
 352

 31
 117

 12

 18

 153
 135

 8266
 1128

 13951
 937

 29

 90

 26

 228
 223

 430
 353

 32

 4

 6554
 138

 11

 530
 330

 0
 17

 0
 52

 120
 135

 85

 24
 129

 11

 0
 6

 6

 6

 849
 178

 849
 178

 5819
 1559

 5819
 1559

 3022
 3817

 3022
 3817

 8

 1876
 1981

 14

 13
 6

 5

 51
 130

 11
 41

 6

 11

 1027
 1636

 0
 19

 0
 4

 7

 7

 4

 3

 151
 19

 151
 19

 151
 19

 143742
 60415

 23643
 28137

 2046
 896

 38
 32

 3818
 1388

 17081
 24953

 660
 868

 119286
 32097

 10
 13

 13

 102726
 14696

 11470
 12162

 7

 722
 884

 90
 123

 3822
 4021

 9
 5

 95
 108

 71

 23

 186
 50

 0
 8

 813
 181

 813
 181

 7433
 6143

 7433
 6143

 1362
 392

 67
 64

 117
 67

 7
 62

 7

 2155
 3169

 3609
 2339

 31
 22

 78

 0
 28

 987
 591

 987
 591

 4

 983
 591

 225
 153

 75
 54

 53
 54

 11

 11

 27
 27

 27
 27

 61
 8

 61
 8

 59
 37

 7
 4

 11

 18

 23

 0
 33

 3
 27

 3
 27

 7117
 2882

 7117
 2882

 7117
 2882

 0
 43

 0
 43

 0
 43

 482
 8779

 477
 8779

 252
 2372

 239
 15

 13
 2

 0
 2213

 0
 136

 0
 2

 178
 6064

 176
 6064

 2

 24
 293

 3
 43

 13
 148

 8
 11

 0
 34

 0
 57

 19
 50

 19
 50

 5

 5

 5

 118
 277

 118
 277

 118
 277

 118
 277

 185
 174

 185
 174

 185
 174

 185
 174

 9126
 17228

 1951
 4910

 807
 2907

 807
 2907

 296
 578

 296
 578

 7065
 12187

 1808
 5094

 1808
 5094

 4983
 6611

 810
 2285

 8

 1584
 149

 1101
 2383

 673
 513

 274
 482

 274
 482

 47

 47

 47

 46
 43

 46
 43

 46
 43

 273
 364

 273
 364

 273
 364

 273
 364

 2

 2

 2

 2

 2

 568
 1013

 452
 732

 452
 732

 452
 732

 452
 732

 98
 237

 98
 237

 94
 237

 82
 187

 12
 50

 4

 4

 4
 12

 4
 12

 2
 3

 2
 3

 2
 9

 2
 9

 7

 7

 7

 7

 34079
 69400

 33980
 69034

 2034
 718

 81
 129

 81
 129

 5

 3

 2

 74
 42

 9
 8

 65
 31

 0
 3

 2
 2

 2
 2

 3

 255
 61

 255
 61

 1550
 398

 327
 16

 1208
 351

 15
 27

 0
 4

 13

 13

 25
 31

 3
 11

 8
 6

 4
 14

 13
 45

 13
 2

 0
 13

 0
 30

 3

 3

 10
 6

 10
 6

 0
 2

 0
 2

 0
 2

 0
 2

 10

 10

 10

 8486
 25136

 8
 46

 3
 25

 5

 0
 21

 55
 282

 26
 106

 29
 11

 0
 20

 0
 145

 6652
 18599

 81
 226

 6145
 16992

 8

 16
 56

 4

 398
 1325

 287
 1302

 16
 585

 30
 37

 18
 198

 0
 18

 0
 30

 3

 3

 6

 6

 1384
 4621

 393
 2231

 3
 18

 52
 25

 2

 61
 6

 4

 5

 801
 1941

 61
 312

 0
 10

 61

 61

 22
 194

 22
 194

 0
 58

 0
 58

 11976
 12366

 2
 6228

 2
 6161

 0
 67

 22
 18

 7

 3
 3

 12
 13

 0
 2

 418
 2913

 32

 306
 2791

 11
 18

 69
 37

 0
 30

 0
 37

 11527
 3033

 191
 6

 7868
 287

 123

 2559
 2270

 783
 450

 0
 20

 7
 162

 7
 162

 0
 12

 0
 12

 2823
 14028

 6
 7

 6
 7

 2775
 13505

 83
 102

 71
 28

 11
 22

 141
 222

 275
 3276

 11
 31

 115
 205

 27
 74

 6

 4

 6
 18

 7

 65
 194

 48
 135

 8
 8

 48
 40

 13
 69

 24
 50

 158
 14

 60
 114

 13
 79

 1422
 7801

 0
 21

 0
 6

 42
 516

 5
 142

 5

 6
 28

 8632
 16558

 2
 14

 2
 14

 507
 282

 507
 282

 7
 18

 7
 18

 4

 4

 9
 21

 9
 21

 8066
 16196

 474
 1985

 7

 6520
 10830

 11

 845
 2695

 209
 556

 0
 3

 0
 6

 0
 11

 0
 15

 0
 46

 37
 2

 37
 2

 0
 17

 0
 17

 0
 8

 0
 8

 19
 206

 19
 206

 19
 206

 0
 22

 0
 22

 0
 22

 12
 2

 12
 2

 12

 12

 0
 2

 0
 2

 19
 89

 19
 89

 19
 89

 19
 89

 12

 12

 12

 12

 51
 213

 51
 213

 51
 213

 26
 114

 25
 97

 2
 52

 2
 52

 2
 52

 2
 52

 3
 10

 3
 10

 3
 10

 3
 10

 906
 1043

 357
 389

 122
 128

 122
 128

 3
 6

 116
 122

 3

 235
 261

 235
 261

 212
 191

 23
 70

 546
 654

 458
 311

 458
 311

 458
 311

 88
 335

 59
 273

 59
 261

 0
 12

 0
 8

 0
 8

 3

 3

 3

 3

 177
 15

 177
 15

 177
 15

 38
 2

 8

 4

 23
 2

 125
 13

 125
 13

 5

 3

 2

 9

 9

 53
 53

 10
 10

 10
 10

 10
 10

 10
 10

 43
 41

 43
 41

 43
 41

 43
 41

 0
 2

 0
 2

 0
 2

 0
 2

 27

 27

 27

 27

 27

 3

 3

 3

 3

 3

 4465
 2904

 4465
 2904

 3819
 2870

 2859
 2492

 2854
 2492

 960
 378

 960
 378

 646
 34

 646
 34

 61
 6

 585
 28

 9
 2

 9
 2

 9
 2

 4
 2

 4
 2

 5

 5

 226
 104

 9
 2

 9
 2

 9
 2

 9
 2

 9
 9

 9
 9

 9

 9

 0
 9

 0
 9

 4
 48

 4
 48

 4
 48

 4
 48

 3
 9

 3

 3

 3

 0
 5

 0
 5

 0
 5

 0
 4

 0
 4

 0
 4

 2

 2

 2

 2

 2

 2

 2

 11
 44

 8
 5

 8
 5

 8
 5

 8
 5

 3
 32

 3
 32

 3
 32

 3
 32

 0
 7

 0
 7

 0
 7

 0
 7

 33
 24

 33
 24

 33
 24

 33
 24

 25
 24

 243546
 99298

 183854
 91112

 6
 6

 6
 6

 2
 4

 4

 0
 2

 112
 155

 112
 155

 4

 108
 155

 7358
 52515

 6222
 51978

 3006
 121

 2701
 14592

 15

 17

 253
 349

 9

 77

 17
 12

 127
 36891

 0
 13

 5

 5

 832
 387

 17
 45

 30

 103
 45

 172
 18

 39
 8

 7

 24

 6
 6

 138
 29

 296
 236

 299
 133

 299
 133

 0
 17

 0
 17

 24
 1081

 24
 1081

 24
 1081

 31
 176

 31
 176

 31
 176

 1874
 83

 38
 3

 38

 0
 3

 1836
 80

 260
 80

 1576

 304
 58

 304
 58

 304
 58

 96833
 8865

 1008
 130

 327
 73

 533
 18

 65

 70

 4
 27

 108
 134

 7
 45

 8

 93
 18

 0
 51

 0
 20

 166
 370

 166
 370

 30021
 2284

 30021
 2284

 7678
 472

 10

 7168
 186

 500
 286

 18
 14

 7

 11
 14

 48640
 4800

 4

 7818
 2084

 40818
 2716

 9194
 661

 9194
 661

 2
 10

 2
 10

 2

 0
 10

 2853
 2519

 2853
 2519

 7

 2696
 2477

 4

 146
 37

 0
 5

 70841
 14719

 2812
 29

 2812
 29

 68029
 14690

 11

 87
 20

 140
 24

 6

 16

 85
 77

 67339
 14390

 345
 179

 133
 212

 133
 212

 133
 212

 237
 23

 237
 23

 4

 17

 23

 36
 5

 9

 85
 18

 42

 15

 3243
 10690

 3243
 10690

 3243
 10690

 49895
 7803

 4

 4

 4

 82
 65

 3

 3

 28
 5

 11

 10
 2

 7

 0
 3

 51
 60

 51
 60

 5
 4

 5
 4

 5
 4

 1483
 696

 17

 17

 1461
 696

 1006
 591

 187
 8

 24

 3

 6

 13
 3

 4

 4

 49

 165
 94

 6

 6

 6

 13
 15

 13
 15

 13
 15

 37253
 5215

 37253
 5215

 11

 8
 788

 21
 897

 21

 90
 318

 6

 51
 51

 119
 2393

 3

 5

 4
 2

 2

 34
 6

 9
 98

 34

 51
 91

 20
 7

 6

 10

 5

 51
 19

 8

 4

 9

 24
 4

 9

 27

 13

 9

 42
 6

 36328
 74

 213
 435

 0
 13

 0
 6

 0
 7

 303
 534

 17

 17

 13

 13

 4
 86

 4
 86

 59
 70

 3
 6

 28
 15

 28
 12

 0
 37

 199
 378

 5

 122
 42

 7

 3

 49
 292

 13

 0
 13

 0
 20

 0
 11

 11

 11

 22

 22

 22

 10724
 1270

 221
 492

 20
 78

 28
 51

 9

 5

 77
 344

 65

 17
 19

 1447
 502

 2

 16

 16
 8

 11
 27

 2
 14

 143
 17

 91

 342
 61

 819
 372

 9040
 263

 1822
 40

 205

 445
 9

 2805
 42

 7

 3

 237

 117
 108

 3373
 55

 6

 12

 8
 5

 0
 4

 4
 13

 4
 13

 12

 12

 0
 4

 0
 4

 0
 4

 21

 13

 13

 4

 9

 2

 2

 2

 6

 6

 6

 910
 48

 7

 7

 7

 903
 48

 45

 16

 29

 7

 2

 3

 2

 851
 48

 339
 19

 112

 27
 5

 373
 24

 11
 4

 11

 11

 11

 0
 4

 0
 4

 0
 4

 8855
 296

 8855
 296

 8855
 296

 8855
 296

 0
 29

 0
 29

 0
 25

 0
 25

 0
 4

 0
 4

 0
 3

 0
 3

 0
 3

 0
 3

 0
 3

 0
 3

 0
 3

 711
 122

 711
 122

 711
 122

 489
 120

 482
 120

 7

 222
 2

 193
 2

 4

 25

 1144
 5078

 913
 4522

 913
 4522

 913
 4522

 313
 1426

 30
 268

 38
 427

 153
 225

 153
 225

 153
 225

 31
 89

 57
 32

 65
 104

 46
 153

 46
 153

 46
 153

 46
 153

 32
 101

 32
 101

 32
 101

 32
 101

 0
 9

 0
 9

 0
 9

 0
 9

 0
 68

 0
 68

 0
 68

 0
 68

 3313
 6282

 5
 30

 5
 30

 5
 30

 5
 30

 156
 139

 49
 44

 49
 44

 60
 58

 60
 58

 4
 4

 31
 19

 14
 2

 14
 2

 14
 2

 33
 11

 33
 11

 33
 11

 0
 4

 0
 4

 0
 4

 0
 18

 0
 18

 0
 18

 103
 278

 99
 270

 99
 270

 99
 270

 4
 8

 4
 8

 4
 8

 79
 299

 79
 299

 79
 299

 79
 299

 955
 2532

 468
 1913

 9
 495

 9
 495

 43
 403

 25
 107

 12
 112

 6
 102

 0
 41

 0
 41

 343
 961

 43
 133

 118
 179

 73
 54

 73
 54

 89
 89

 86
 84

 86
 84

 3

 3

 0
 5

 0
 5

 383
 520

 11
 6

 11
 6

 372
 514

 372
 514

 15
 10

 15
 10

 15
 10

 541
 406

 541
 406

 541
 406

 541
 406

 533
 1462

 533
 1462

 533
 1462

 533
 1462

 46
 10

 29
 7

 29
 7

 29
 7

 17
 3

 6
 3

 6
 3

 11

 7

 4

 224
 163

 224
 163

 224
 163

 224
 163

 410
 890

 410
 890

 410
 890

 410
 890

 261
 73

 261
 73

 261
 73

 261
 73

 52
 36

 52
 36

 52
 36

 52
 36

 52
 36

 14
 59

 14
 59

 14
 57

 12
 37

 12
 37

 2
 20

 2
 20

 0
 2

 0
 2

 0
 2

 6167
 6992

 42
 161

 42
 161

 42
 161

 42
 161

 3857
 2603

 3857
 2603

 57
 56

 57
 56

 3782
 2437

 3
 25

 6

 29

 97
 186

 3600
 2198

 0
 8

 18
 110

 18
 110

 2268
 4228

 25
 121

 25
 121

 25
 121

 334
 684

 334
 684

 334
 684

 65
 77

 65
 77

 65
 77

 495
 780

 495
 780

 495
 780

 23
 65

 23
 65

 21
 49

 0
 5

 1310
 2464

 128
 242

 128
 242

 30
 191

 30
 191

 808
 1041

 72
 70

 567
 541

 169
 175

 0
 95

 0
 12

 0
 140

 184
 908

 8
 17

 27
 79

 160
 57

 160
 57

 0
 21

 0
 21

 0
 4

 0
 4

 2

 2

 2

 14
 37

 14
 37

 14
 37

 3
 64

 3
 64

 3
 64

 3
 64

 3
 64

 235
 351

 235
 351

 235
 351

 235
 351

 235
 351

 2446
 2853

 2

 2

 2

 2

 2444
 2845

 2444
 2845

 88
 180

 88
 180

 6
 2

 6
 2

 698
 654

 2

 183
 21

 345
 520

 168
 71

 0
 42

 1652
 1975

 1652
 1975

 0
 34

 0
 34

 0
 6

 0
 6

 0
 6

 0
 6

 0
 2

 0
 2

 0
 2

 0
 2

 1217
 790

 2

 2

 2

 2

 895
 10

 82

 82

 82

 813
 10

 813
 10

 3

 168

 372
 6

 270
 4

 320
 780

 3
 8

 3
 8

 3
 6

 0
 2

 268
 685

 268
 685

 8
 27

 33
 17

 220
 541

 0
 12

 32
 15

 32
 15

 18

 14
 5

 0
 2

 0
 2

 0
 6

 15
 72

 9
 65

 9
 65

 2

 2

 2

 1198236
 840716

 208662
 403450

 39740
 118372

 39740
 118372

 19

 101
 38

 1985
 7372

 40
 44

 48
 200

 21
 176

 32
 41

 183
 99

 863
 8273

 4

 13
 81

 809
 2841

 11
 30

 16
 158

 43
 43

 1092
 1436

 113
 54

 34015
 96987

 0
 48

 0
 7

 3

 3

 3

 515
 587

 506
 573

 159
 44

 3

 331
 529

 9

 9
 9

 9
 9

 7
 46

 7
 46

 7
 46

 7077
 13327

 6946
 12796

 14
 70

 2335
 3351

 332
 1275

 1203
 630

 11
 8

 2890
 7266

 112
 531

 36
 284

 63
 242

 19

 19

 79
 110

 74
 110

 74
 110

 19
 25

 19
 25

 19
 25

 165
 69

 0
 5

 21

 21

 39
 42

 10

 3
 6

 3
 6

 126
 880

 126
 880

 18
 152

 0
 39

 123

 123

 123

 61
 42

 61
 42

 49

 0
 42

 41
 95

 41
 95

 41
 10

 0
 85

 1974
 262

 1974
 262

 1330
 262

 115192
 152372

 145
 69

 145
 69

 62194
 110619

 14883
 78379

 24
 13

 902
 834

 339
 422

 3
 5

 36

 42016
 13221

 2348
 16320

 222
 49

 25

 57
 22

 915
 673

 183
 65

 125
 585

 136
 35

 136
 35

 2593
 3139

 2325
 2716

 268
 316

 0
 103

 568
 820

 159

 146
 391

 173
 229

 72
 176

 11
 23

 11
 23

 14
 70

 14
 70

 37
 143

 37
 143

 467
 796

 25
 8

 152
 36

 138
 36

 14

 2315
 1512

 29
 29

 2239
 1476

 47
 7

 36754
 17247

 241
 93

 7059
 2428

 42

 25843
 11137

 71
 68

 71

 5

 7

 6

 13
 19

 38
 236

 209
 256

 13
 42

 284
 4

 2724
 2920

 19
 32

 0
 9

 0
 3

 452
 762

 49
 10

 59
 94

 222

 0
 130

 8
 120

 8
 120

 30
 5

 30

 0
 5

 5795
 8684

 83
 209

 11
 28

 1983
 5141

 8

 99
 299

 131
 270

 39

 183
 444

 341
 61

 15
 4

 64
 114

 17
 59

 4

 2279
 1434

 0
 6

 3433
 8184

 3433
 8184

 0
 19

 0
 19

 6773
 7403

 6773
 7403

 849
 1754

 71
 63

 95
 334

 36

 6
 9

 2

 18

 3861
 2274

 155
 31

 15

 711
 1318

 896
 1485

 7
 6

 10
 11

 0
 12

 0
 13

 42
 4

 27

 10

 5

 32726
 106572

 32726
 106572

 469
 787

 41

 6

 205
 363

 7
 48

 5
 60

 759
 1279

 125
 107

 220
 198

 100
 363

 10

 12
 32

 2185
 1249

 5

 25466
 95009

 257
 27

 120
 440

 2720
 6544

 135
 414

 135
 414

 55
 319

 5
 3

 11

 3466
 2781

 3466
 2781

 3466
 2781

 0
 4

 0
 4

 0
 4

 0
 17

 0
 17

 0
 17

 0
 2

 0
 2

 0
 2

 0
 3

 0
 3

 0
 3

 989344
 437118

 519

 519

 519

 800
 36

 793
 36

 774
 36

 19

 7

 7

 4001
 49

 424
 45

 45
 4

 363
 16

 16
 25

 3532

 3532

 36

 36

 9
 4

 9
 4

 53

 53

 107869
 82780

 7
 28

 7
 28

 387
 1988

 12
 12

 9

 14

 12

 9

 11
 60

 320
 1910

 0
 3

 0
 3

 2911
 2908

 637
 1056

 95
 63

 442
 53

 58

 63
 547

 14

 1470
 180

 132
 1009

 53478
 39456

 124
 537

 31232
 712

 8
 125

 27

 340
 43

 1745
 22801

 970
 14

 12
 56

 7

 8

 10
 124

 158
 680

 23
 190

 129
 411

 29
 26

 112
 25

 6
 17

 22
 12

 31

 8767
 170

 1100
 5514

 12

 8574
 7760

 0
 14

 0
 16

 0
 37

 15

 6

 9

 5388
 400

 3

 5333
 337

 46
 9

 6
 54

 6

 6

 36
 10

 26
 7

 4
 3

 6

 395
 195

 151
 14

 228
 108

 16
 73

 5
 22

 5
 22

 10344
 620

 3

 24

 58

 1789
 125

 9
 397

 237
 36

 117
 1007

 22

 4
 664

 38
 23

 9
 140

 19
 59

 25
 119

 0
 2

 32508
 31642

 19
 307

 620
 1765

 966
 6492

 8

 4856
 206

 9376
 16042

 235
 210

 44

 4420
 63

 11959
 6518

 0
 2

 0
 15

 964
 609

 10

 7

 92

 96

 135

 41
 9

 10
 49

 164
 23

 409
 466

 0
 62

 320
 1586

 320
 1586

 65
 10

 65

 0
 10

 361
 144

 361
 144

 562
 2083

 562
 2083

 0
 72

 0
 72

 74

 74

 74

 87
 361

 87
 361

 87
 361

 5
 59

 5

 5

 0
 31

 0
 31

 0
 28

 0
 28

 14
 13

 14
 13

 14
 13

 420
 873

 420
 873

 117
 12

 194
 812

 56
 14

 15
 2

 169128
 193873

 226
 9881

 226
 9881

 1156
 214

 157
 89

 301
 66

 175

 523
 59

 128812
 99739

 105939
 47708

 25
 669

 2203
 7728

 20645
 43634

 347
 24948

 5

 332
 24496

 3

 7

 0
 301

 0
 151

 6638
 9232

 4363
 8783

 2188
 444

 87
 5

 2261
 3682

 44

 113
 4

 2104
 3604

 0
 74

 29688
 46177

 29688
 46177

 230
 931

 230
 931

 161
 916

 59

 10
 7

 0
 8

 7
 9

 7
 9

 7
 9

 177
 11

 177
 11

 177
 11

 8

 8

 8

 9

 9

 9

 40
 824

 7
 798

 7
 94

 0
 213

 0
 491

 33

 33

 0
 26

 0
 26

 93
 2717

 93
 2717

 55
 538

 38
 2140

 0
 33

 5593
 118

 5593
 118

 3897

 198

 12

 1486
 118

 101
 351

 101
 351

 101
 351

 661542
 105106

 75485
 36074

 54520
 34289

 45
 11

 11

 13178
 236

 196
 5

 954
 695

 6578
 630

 586057
 69032

 114
 13

 585943
 68996

 0
 23

 56
 443

 56
 443

 56
 443

 144
 77

 144
 77

 100
 70

 33

 0
 7

 128
 494

 128
 494

 65
 92

 40
 142

 621

 621

 621

 9
 166

 9
 166

 9
 166

 6765
 12912

 1016
 2822

 25

 984
 2798

 0
 4

 5727
 9686

 139
 848

 89

 77
 31

 141
 9

 2125
 2382

 52

 3104
 6416

 22
 404

 22
 404

 30851
 34906

 30851
 34906

 30851
 34906

 0
 9

 0
 9

 0
 9

 230
 148

 230
 148

 230
 148

 230
 148

 44
 49

 44
 49

 44
 49

 44
 49

 44
 49

 10
 38

 10
 38

 10
 38

 10
 38

 10
 38

 35
 6

 35
 6

 35
 6

 35
 6

 35
 2

 0
 4

 3
 2

 3
 2

 3

 3

 3

 0
 2

 0
 2

 0
 2

 14
 13

 14
 13

 14
 13

 14
 13

 14
 13

 3

 3

 3

 3

 3

 1315
 1698

 738
 146

 738
 146

 11

 8

 3

 18
 4

 18
 4

 2

 2

 587
 114

 4

 8
 3

 413
 16

 18
 7

 84
 52

 0
 24

 76
 8

 46
 8

 3

 4
 10

 4
 10

 40
 10

 40
 10

 6
 26

 6
 26

 6
 26

 6
 26

 58

 58

 44

 44

 14

 14

 513
 1526

 208
 839

 151
 548

 87
 272

 46
 207

 18
 61

 0
 8

 49
 150

 47
 119

 2
 31

 8
 65

 8
 65

 0
 49

 0
 49

 0
 27

 0
 27

 6
 11

 6
 11

 79
 197

 79
 197

 2
 5

 8
 22

 5

 8

 9

 41
 96

 0
 22

 213
 439

 213
 436

 20
 25

 164
 53

 12
 66

 5
 12

 0
 3

 0
 3

 7
 15

 7
 15

 7
 15

 0
 25

 0
 25

 0
 25

 18
 110

 18
 110

 18
 110

 18
 110

 18
 110

 87

 87

 87

 87

 87

 0
 14

 0
 14

 0
 14

 0
 14

 0
 14

 0
 11

 0
 11

 0
 11

 0
 11

 0
 11

 0
 3

 0
 3

 0
 3

 0
 3

 0
 3

 0
 3

 0
 3

 0
 3

 0
 3

 0
 3
