# Supplementary material for: Fungi are more transient than bacteria in caterpillar gut microbiomes
Source: Sci Rep. 2022 Sep 16;12:15552. doi: 10.1038/s41598-022-19855-5 (PMC9481635; doi:10.1038/s41598-022-19855-5)
Supplement: Supplementary file 2 — Supplementary Figure S2. [file 41598_2022_19855_MOESM2_ESM.html]

Javascript must be enabled to view this page.

magnitude

guts
leaves

 4847536
 1231450

 4847536
 1231450

 4657208
 1190250

 1413
 354

 2

 1411
 354

 1400
 354

 1400
 354

 23
 33

 4080962
 1050659

 2

 2

 2

 2

 837
 17

 834
 17

 513
 11

 321
 6

 321
 6

 3

 3

 26216
 3042

 22566
 2383

 193
 64

 89
 13

 104
 51

 15704
 1315

 128
 47

 7

 12
 388

 799
 200

 294
 73

 2

 3487
 656

 3487
 656

 3487
 656

 518972
 299342

 3

 3

 3

 6845
 2622

 5033
 1063

 1189
 110

 1709
 1534

 690
 58

 85
 25

 85
 7

 0
 15

 0
 3

 18

 18

 19947
 1542

 10826
 976

 10760
 934

 6138
 486

 51

 42

 19

 19

 733
 239

 301
 3

 428
 150

 428
 150

 4

 4

 0
 86

 0
 86

 1513
 152

 1190
 65

 31
 23

 31
 23

 190
 27

 190
 27

 402082
 259111

 28

 22

 86
 18

 40
 18

 46

 6

 6

 211
 229

 211
 229

 537
 27

 537
 27

 2

 131
 19

 2

 129
 19

 26
 37

 6

 9
 5

 0
 30

 0
 2

 42

 42

 725
 236

 27

 34
 10

 13
 21

 13
 21

 384655
 255306

 89

 85

 1638
 97

 68
 7

 151
 8

 133294
 106398

 0
 20

 27

 27

 2874
 221

 0
 2

 746
 141

 59
 53

 380
 44

 0
 5

 77
 12

 4

 1774
 60

 50

 471
 19

 812
 10

 773
 10

 22

 689
 4

 0
 2

 2

 2

 2

 28980
 6775

 244
 6

 57
 2

 187
 4

 808
 324

 775
 324

 153
 20

 153
 20

 269
 52

 30

 230
 52

 1097
 202

 1097
 202

 2033
 159

 4

 1120
 149

 250
 253

 142
 47

 48
 41

 22

 22

 332
 121

 332
 121

 528
 109

 157

 157

 2876610
 642706

 2352451
 468106

 2352451
 468106

 2352451
 468106

 165
 63

 111

 111

 36

 36

 13

 13

 5
 2

 5
 2

 3

 3

 3

 499662
 163803

 469283
 155507

 9

 259
 48

 590
 161

 8382
 371

 3901
 2816

 3901
 2816

 6565
 564

 5435
 531

 7312
 1223

 7312
 1223

 1618
 227

 1618
 227

 583
 4

 583
 4

 791
 210

 789
 186

 2
 24

 244
 6

 244
 6

 0
 7

 6
 8

 3
 2

 7665
 1751

 4697
 1268

 1001
 167

 25
 9

 32

 32

 526
 85

 526
 85

 526
 85

 1918
 199

 252
 74

 64
 70

 188
 4

 1382
 75

 1382
 75

 135
 38

 135
 38

 135
 38

 135
 35

 0
 3

 2

 2

 2

 2

 610785
 98431

 6962
 457

 6962
 457

 6962
 457

 68
 8

 68
 8

 59

 0
 8

 826
 269

 826
 269

 6310
 378

 6173
 376

 64
 5

 214
 9

 12

 12

 73445
 4185

 68948
 3961

 24942
 617

 10474
 809

 1416
 106

 1407
 106

 9

 25

 25

 438
 30

 4
 3

 1078
 2

 933

 145
 2

 599
 14

 4

 4

 595
 14

 134
 10

 459
 4

 182443
 44227

 39

 414
 262

 414
 262

 72

 49

 23

 63
 11

 63

 7257
 200

 280
 7

 3959
 103

 2880
 69

 11

 127
 21

 25367
 1402

 2855
 13

 2855
 13

 174

 4

 170

 1985
 389

 733
 55

 625
 24

 88
 3

 7
 4

 15857
 105

 15857
 105

 4

 0
 10

 0
 10

 3517
 835

 3517
 835

 99
 4

 99
 4

 99
 4

 2202
 26

 109

 93

 23

 3

 20

 348
 7

 348
 7

 1449
 17

 71

 68

 23133
 1311

 18770
 6

 18770
 6

 3803
 1166

 236
 9

 149

 3361
 756

 0
 384

 4

 4

 107
 79

 14
 43

 26

 26

 130

 56

 74

 0
 5

 0
 5

 0
 3

 0
 3

 1397
 88

 1277
 88

 74

 180
 52

 157
 9

 7

 113

 632
 11

 632
 11

 577
 11

 4

 51

 23115
 3469

 11

 11

 6

 6

 58
 2

 58
 2

 22973
 3456

 13903
 3324

 28
 30

 1092

 4858
 332

 428
 20

 17
 69

 17
 69

 136
 58

 136
 58

 8

 8

 2262
 144

 2262
 144

 3
 6

 3
 6

 1979
 16

 227
 11

 14

 3

 3

 3

 2317
 189

 2317
 189

 18

 1727
 60

 1727
 60

 253
 19

 160
 2

 42

 155
 9

 54917
 8757

 299
 19

 299
 19

 934

 934

 204

 2586
 450

 1138
 254

 13692
 2878

 13692
 2878

 14249
 1028

 72

 780
 188

 12987
 799

 224
 23

 2

 128
 2

 128
 2

 491
 126

 3

 1497
 118

 1111
 35

 22

 5031
 613

 742
 90

 140
 68

 284
 14

 1059
 152

 4
 3

 20
 4

 20
 4

 489
 173

 56

 10
 75

 423
 98

 1093
 22

 1093
 22

 173
 152

 103
 144

 1228
 127

 1147
 127

 9

 72

 97
 27

 33

 33

 2

 1035
 72

 1035
 70

 1035
 42

 0
 28

 130122
 15216

 118893
 14567

 64701
 10560

 1469
 72

 390

 81

 12

 0
 25

 3106
 32

 555
 32

 2551

 1096

 1334
 18

 15

 36

 15

 1268

 0
 9

 3708
 197

 1764
 8

 268
 20

 1409
 155

 1025
 368

 936
 360

 8

 8

 0
 10

 7026
 3785

 695
 3

 695
 3

 246

 246

 2

 2

 49
 3

 49
 3

 2180
 111

 18

 209
 189

 209
 189

 64

 64

 3581
 3475

 232

 232

 2

 2

 2

 100
 133

 97
 83

 82
 6

 10

 5

 0
 16

 3
 50

 0
 39

 0
 11

 3382
 50

 238
 12

 126
 9

 31

 81

 32

 32

 32

 1260
 23

 151
 4

 32
 4

 119

 963
 13

 113

 1397

 180

 180

 1217

 42

 917

 192

 3

 0
 6

 0
 6

 0
 6

 753
 53

 404
 46

 399
 40

 2

 336
 7

 336
 7

 23

 23

 23

 23

 9176
 1112

 1772
 465

 338
 366

 12

 4

 322
 366

 71
 6

 9

 86

 53

 31

 1271
 93

 4044
 145

 25
 7

 25
 3

 12

 12

 34
 3

 34
 3

 3801
 133

 167
 4

 39
 4

 63
 7

 68

 2
 5

 0
 6

 0
 6

 0
 6

 0
 6

 103107
 22205

 1762
 19

 1566

 1566

 1566

 3653
 40

 3316
 38

 1779
 7

 186

 710

 7

 14

 490
 4

 65

 1489
 31

 7

 27

 133
 6

 662
 12

 46

 22

 23

 4

 78
 13

 12

 19

 138

 5

 69

 337
 2

 333

 10

 3

 3

 2

 6

 21

 9

 24

 10

 4
 2

 4
 2

 91081
 21116

 2660
 116

 22

 22

 2638
 110

 2

 13

 219
 15

 28

 2426
 187

 2426
 187

 2426
 187

 4684
 357

 50
 8

 5

 2005
 220

 12

 224
 12

 131
 12

 894
 53

 5

 189

 9

 17
 6

 3

 2
 8

 220
 17

 10

 4

 0
 2

 0
 7

 2
 2

 4

 4

 42
 21

 11
 21

 31

 247
 15

 26

 26

 5256
 1590

 29
 17

 29
 17

 4186
 781

 4186
 781

 1041
 792

 1041
 792

 1765
 444

 1741
 442

 24
 2

 24
 2

 42081
 8295

 85

 85

 1611
 95

 72
 14

 194

 20

 21308
 1881

 3118
 65

 2

 0
 15

 0
 9

 15869
 5432

 9940
 2552

 506
 324

 555
 103

 25

 25

 526
 103

 2
 13

 2

 0
 13

 524
 90

 215
 10

 3476
 314

 1877
 262

 4

 4

 2

 2

 9

 9

 21
 5

 21
 5

 13

 2
 5

 103100
 17392

 7

 7

 7

 7

 5435
 1825

 8
 77

 8
 37

 8
 37

 0
 40

 0
 40

 5427
 1748

 1655
 194

 1550
 191

 103
 3

 2

 3772
 1544

 2115
 907

 184

 0
 10

 0
 10

 1375
 53

 1375
 53

 365
 13

 77043
 14010

 17
 10

 17
 10

 17
 10

 21
 10

 21
 10

 0
 10

 61621
 11869

 154
 34

 9

 6
 16

 123
 18

 11

 21

 21

 65
 472

 53373
 10359

 53373
 10359

 0
 2

 0
 2

 104
 21

 104
 21

 102
 11

 568
 283

 436
 111

 431
 85

 5
 26

 132
 46

 132
 46

 0
 58

 0
 58

 0
 65

 0
 34

 0
 22

 0
 3

 0
 3

 96

 96

 96

 6244
 400

 6244
 400

 6240
 400

 3368
 104

 392
 51

 22

 2

 62
 8

 17

 2976
 53

 126

 2308
 28

 542
 25

 24

 24

 24

 17306
 1259

 17306
 1259

 17206
 1251

 17206
 1251

 96
 8

 65
 8

 1198
 204

 1193
 204

 1193
 204

 1193
 204

 4

 4

 4

 4

 328
 10

 328
 10

 275
 2

 5

 2

 95
 2

 34
 8

 34
 8

 5

 5

 5

 5

 0
 13

 0
 13

 0
 13

 102655
 52633

 22070
 30108

 22070
 30108

 3463
 4519

 3463
 4519

 16788
 24952

 3

 146

 150

 9

 13

 20

 0
 14

 343
 2

 53
 2

 68

 864
 278

 160

 412
 215

 57

 116
 164

 57
 7

 13

 0
 58

 0
 17

 0
 41

 72372
 17936

 57

 57

 57

 25801
 5057

 10075
 1391

 2036
 1169

 3

 44

 16
 12

 3

 0
 12

 11601
 3176

 6659
 2157

 665
 156

 665
 156

 282
 16

 282
 16

 427
 48

 208
 25

 109

 4

 5

 5

 649
 130

 630
 130

 19

 44

 14

 30

 0
 2

 1818
 508

 6
 19

 6
 19

 429
 201

 371
 170

 10
 3

 2

 182
 12

 161
 12

 10

 10

 10

 10

 25
 20

 0
 20

 34
 54

 3

 31
 31

 2

 124
 7

 620
 123

 161

 147

 3

 32

 7

 0
 19

 0
 19

 0
 39

 0
 39

 0
 9

 1066
 189

 398
 34

 390
 52

 0
 3

 11

 11

 3
 21

 3
 2

 9
 4

 12

 5

 5

 127

 127

 4

 4

 21

 0
 17

 0
 7

 0
 7

 0
 2

 0
 2

 0
 2

 0
 2

 3710
 248

 2461
 102

 67
 84

 59
 8

 5

 5

 18

 18

 3

 3

 169
 10

 163
 6

 0
 4

 51

 2

 4

 12

 2

 3

 119

 2

 218
 9

 431
 43

 431
 43

 143

 143

 12

 26
 8

 12
 6

 12
 6

 14
 2

 14
 2

 33
 57

 6
 48

 0
 33

 11

 11

 16

 22
 22

 19

 111
 22

 111
 22

 33760
 11149

 16669
 1022

 16669
 1022

 5120
 1287

 5120
 1287

 151
 353

 151
 353

 1477
 83

 1162
 83

 315

 127

 116

 4277
 312

 46
 13

 0
 2

 0
 2

 17
 9

 17
 9

 17
 9

 3606
 144

 3526
 134

 1313
 54

 3

 0
 4

 80
 10

 80
 10

 414
 111

 354
 85

 11

 11

 34
 4

 34
 4

 1139
 2412

 1139
 2412

 906
 2366

 57
 7

 57
 7

 160
 39

 31
 8

 77
 11

 52
 20

 5826
 1833

 3243
 1821

 40

 40

 73

 81
 5

 29
 5

 44
 4

 44
 4

 44
 4

 848
 226

 848
 222

 67
 92

 31

 3
 20

 3

 0
 20

 33
 72

 6
 22

 14
 40

 93
 4

 93
 4

 93
 4

 638
 36

 638
 36

 2

 2

 2

 4
 17

 0
 5

 0
 5

 37

 37

 595
 4

 19

 19

 576
 4

 576
 4

 0
 15

 0
 15

 0
 15

 2

 2

 2

 2

 2

 21927
 393

 21923
 393

 1787
 5

 1664
 5

 1664
 5

 104

 104

 19

 19

 4

 4

 4

 2975
 196

 2975
 196

 2975
 170

 305
 20

 302

 302

 3
 20

 3

 0
 20

 8
 8

 4

 4
 8

 2
 8

 2

 456
 8

 57
 8

 57
 8

 3

 3

 98

 98

 33

 265

 265

 15562
 35

 15553
 35

 5

 19

 15371
 23

 5
 2

 2

 11

 0
 5

 9

 9

 196

 196

 196

 192034
 35006

 5

 5

 5

 64
 4

 64
 4

 56
 4

 15

 2

 4

 17
 16

 17
 16

 17
 16

 17
 16

 106293
 15193

 155
 2

 155
 2

 778
 249

 2181
 801

 1433
 801

 17
 8

 0
 8

 744

 583
 16

 2
 2

 6

 575
 14

 575
 14

 29720
 9243

 8098
 2253

 8098
 2253

 7
 207

 7
 207

 312
 23

 9228
 1945

 9228
 1945

 19
 95

 19
 50

 11

 11

 231
 57

 10794
 3460

 2925
 423

 3690
 2681

 0
 4

 13

 13

 0
 7

 0
 7

 1036
 27

 872
 14

 700
 9

 5

 2

 146

 6

 27

 27

 113
 13

 113
 13

 6815
 85

 6815
 85

 6744
 85

 77

 77

 77

 75
 2

 50

 50

 25
 2

 25
 2

 63859
 4455

 84

 84

 7674
 244

 7043
 238

 60
 2

 37275
 2367

 63
 4

 20

 74

 7

 8938
 124

 3133
 1025

 294
 1010

 70
 6

 23

 3
 6

 2837
 15

 7

 7

 530
 3

 414

 110
 3

 34

 18

 1987
 9

 837

 10

 279

 71

 10

 198

 0
 3

 2

 2

 2

 39689
 9717

 40
 2

 20
 2

 14
 2

 6

 17

 3

 325
 3

 307

 4

 4

 9

 9

 0
 3

 0
 3

 9827
 4986

 2960
 394

 2953
 394

 334
 6

 39
 6

 295

 424
 3

 424
 3

 5807
 4583

 3242
 4579

 57

 57

 229
 124

 60

 60

 2

 167
 124

 8

 35

 120
 124

 2383
 70

 311
 18

 84
 2

 3

 18

 9

 48
 13

 3

 65

 40
 3

 109
 7

 30

 20

 59
 7

 993
 16

 993
 16

 199
 22

 742
 7

 95

 250

 116
 3

 40
 2

 192

 44

 21180
 4387

 65
 2

 2987
 1962

 315

 1833
 4

 685
 1949

 5
 9

 10

 95

 39

 10

 29

 14378
 2156

 9806
 1411

 312

 1322
 41

 1089
 10

 1920
 212

 1915
 201

 5
 11

 3

 3

 2

 2

 30
 2

 30
 2

 91

 44

 44

 124
 3

 124
 3

 33

 4

 4

 29

 25

 4

 40
 43

 40
 36

 40
 36

 55

 55

 55

 5366
 89

 72

 72

 165

 37

 123

 5078
 78

 5

 51

 51

 0
 4

 668

 668

 2

 2

 166

 115

 418

 409

 73

 14

 7

 7

 2

 2

 222
 76

 222
 76

 4

 4

 216
 76

 2

 2

 26

 26

 26

 226
 23

 43
 23

 0
 23

 0
 23

 183

 71

 2

 2

 110

 328
 31

 328
 31

 328
 31

 196

 33

 3

 9

 8
 31

 36

 18

 10

 10

 39

 39

 39

 11

 28

 10

 10

 10

 10

 2

 2

 2

 2

 2

 2

 2

 453
 113

 28
 2

 4
 2

 4
 2

 24

 24

 182
 39

 4

 4

 4

 4

 30
 2

 0
 2

 23

 23

 7

 7

 33

 33

 0
 33

 0
 33

 0
 4

 0
 4

 96
 72

 6
 2

 6
 2

 38

 52
 70

 0
 10

 0
 60

 84

 23

 23

 23

 27

 27

 20

 7

 509
 36

 509
 36

 509
 36

 33

 209
 24

 209
 24

 209
 24

 177
 11

 0
 5

 39636
 8596

 20293
 6688

 34

 28
 24

 28
 24

 6221
 3119

 5848
 1341

 373
 1739

 10046
 1639

 10046
 1639

 292
 28

 292
 28

 250
 28

 39

 3

 843
 1

 53

 9517
 785

 674
 18

 674
 18

 1061
 32

 59

 999
 32

 2309
 213

 2309
 213

 2410
 149

 63

 136
 60

 55
 16

 2108
 58

 42
 3

 0
 12

 25
 21

 16

 5

 146
 6

 87
 6

 5
 35

 5
 35

 5
 35

 1263
 20

 914

 914

 349
 20

 98
 7

 4

 69
 8

 820
 79

 347
 20

 389
 53

 389
 53

 33

 33

 51
 6

 51
 6

 647
 18

 3

 25

 25

 17

 17

 5

 2

 3

 3

 433

 317

 126
 2

 126
 2

 32

 4

 0
 8

 0
 8

 3589
 93

 2

 2

 27

 2516
 56

 2504
 56

 12

 1026
 37

 18

 18

 0
 38

 0
 38

 0
 38

 3

 3

 3

 3

 3

 190051
 40773

 37408
 8982

 3593
 487

 71
 4

 45

 20

 2

 2

 21

 23

 18

 3

 3

 0
 2

 0
 2

 0
 2

 0
 2

 4

 4

 4

 8

 8

 2

 6

 4

 4

 4

 21

 21

 21

 2

 2

 2

 45
 35

 6
 12

 3

 3

 0
 12

 32
 23

 8

 9

 2

 2

 2

 3
 7

 3

 3

 0
 7

 0
 3

 0
 4

 550
 14

 546

 546

 4
 14

 2
 14

 2

 29
 7

 29
 7

 17
 2

 3

 2458
 367

 2291
 361

 172

 209
 42

 432
 39

 347
 87

 0
 2

 99

 3

 3

 2

 53

 4

 2

 2

 66
 6

 7

 9

 10

 3

 2

 9

 8
 6

 15

 26
 3

 4
 3

 4
 3

 22

 22

 171
 24

 171
 24

 4

 167
 24

 2

 2

 45
 2

 2

 24

 24

 13

 13

 0
 2

 0
 2

 99
 24

 7

 7

 2

 2

 4
 2

 4
 2

 10

 10

 74
 20

 20
 4

 2

 2

 24
 2

 15

 3

 2

 0
 3

 0
 3

 0
 3

 0
 5

 2

 2

 0
 2

 0
 2

 8

 8

 20

 20

 20

 16

 2

 30
 40

 3
 40

 0
 40

 0
 40

 572
 83

 73

 73

 73

 67

 67

 67

 2

 2

 2

 425
 83

 327
 76

 261
 76

 4

 62

 98

 98

 0
 7

 0
 7

 5

 5

 5

 389

 386

 3

 3

 2

 2

 381

 381

 3

 3

 3

 444
 69

 16
 9

 16
 9

 2

 12
 7

 0
 2

 85
 25

 85
 25

 2

 45
 2

 2

 22
 23

 331
 35

 50
 7

 13
 11

 13
 5

 0
 6

 7
 4

 7
 4

 4606
 3363

 41
 105

 15

 12

 2

 2

 12
 2

 5
 2

 6

 0
 103

 0
 103

 4565
 3258

 4565
 3258

 4565
 3258

 86
 12

 86
 12

 29
 4

 2

 6

 21
 4

 57
 8

 39
 8

 18

 749
 170

 39
 2

 2

 2

 14

 2

 2

 10

 17

 17

 6

 0
 2

 184
 12

 64
 2

 18
 2

 46

 28

 28

 92
 10

 27
 4

 65
 6

 523
 156

 137
 98

 15

 55
 73

 67
 25

 58

 58

 2

 2

 323
 42

 2
 2

 13

 102
 18

 7

 199
 19

 0
 3

 3

 3

 3

 6

 6

 6

 6

 10281
 3367

 3

 3

 181
 35

 10
 4

 10
 4

 3

 3

 18
 9

 18
 9

 97
 2

 79
 2

 9

 9

 2

 2

 4

 4

 0
 8

 0
 8

 973
 108

 940
 108

 302
 4

 30

 33

 33

 73

 73

 7

 42
 5

 33

 33

 9

 9

 0
 5

 0
 5

 76

 69

 69

 172

 2

 2

 3

 3

 3

 3

 29

 29

 97

 12

 2

 12

 43

 28

 38

 38

 1859
 1110

 1773
 1096

 1773
 1096

 5

 5

 16
 9

 2

 0
 9

 65

 48

 0
 5

 0
 5

 6861
 2103

 9
 9

 9
 9

 3413
 1216

 18

 3395
 1216

 14
 2

 8
 4

 8
 4

 1996
 355

 1996
 355

 514
 373

 57
 14

 278
 277

 355
 34

 355
 34

 23

 23

 495
 87

 195
 25

 3

 183
 18

 72
 8

 3

 3

 3

 38
 6

 9

 6

 13
 6

 13
 6

 3

 3

 13

 7

 6

 15460
 1306

 6

 6

 6

 581
 14

 581
 14

 174
 2

 13

 13

 3

 10

 21

 8

 2

 2

 2

 4

 2

 2

 14569
 1276

 14419
 1276

 790
 27

 77

 29

 5297
 565

 2
 7

 6

 7461
 635

 303
 23

 0
 3

 2

 2

 2

 236
 14

 225
 14

 219
 14

 6

 0
 2

 0
 2

 0
 2

 2

 2

 2

 145

 145

 3

 3

 142

 142

 253
 50

 179
 3

 23

 23

 15
 3

 15
 3

 14

 14

 124

 32

 7

 38

 8

 64
 47

 64
 47

 32
 47

 27

 2

 769
 88

 433
 69

 48
 11

 48
 11

 149
 36

 5

 236
 22

 191
 10

 15
 4

 0
 2

 45
 12

 2

 6

 6
 12

 5510
 260

 238
 6

 238
 6

 113
 6

 113
 6

 2

 4156
 237

 3898
 227

 3898
 227

 3823
 227

 75

 12
 6

 246
 4

 246
 4

 154
 2

 10

 80
 2

 2

 1053
 17

 134
 8

 134
 8

 134
 6

 11

 11

 11

 9

 9

 2

 33087
 7062

 271
 20

 71
 8

 71
 8

 71
 8

 22392
 4009

 5

 5

 5

 22387
 4009

 22384
 4009

 86
 27

 12886
 3487

 180

 4
 2

 411
 48

 1115
 89

 51

 17

 75
 9

 10424
 3028

 1503
 358

 1503
 358

 1503
 358

 8147
 2664

 8147
 2664

 7435
 1567

 712
 1080

 0
 5

 0
 5

 0
 5

 0
 5

 3

 3

 328

 328

 325

 325

 17

 23

 285

 14765
 3125

 55
 2

 55
 2

 55
 2

 55
 2

 1276
 216

 1276
 216

 982
 209

 14

 16

 36

 18
 3

 3

 4

 4

 4

 2

 13019
 2888

 798
 54

 737
 54

 737
 54

 18

 18

 2

 2

 11

 5

 6

 20

 5

 5

 12221
 2834

 12221
 2834

 12221
 2834

 395
 19

 395
 4

 8

 5

 3

 387
 4

 387
 4

 54
 36

 10
 1

 5
 1

 5
 1

 2
 1

 3

 5

 2

 2

 3

 3

 44
 35

 44
 35

 44
 35

 44
 35

 70

 70

 70

 70

 53

 97478
 21180

 4250
 850

 372
 62

 372
 62

 154
 29

 101
 2

 117
 31

 13

 13

 3082
 785

 1313
 272

 1068
 215

 245
 57

 956
 215

 946
 215

 10

 654
 295

 654
 295

 127

 127

 88794
 20051

 88619
 19838

 88571
 19821

 304

 721
 41

 56679
 11749

 10
 17

 0
 15

 38

 16

 37
 32

 37
 26

 4

 10

 8

 13
 17

 0
 6

 0
 6

 293
 34

 293
 34

 293
 34

 293
 34

 3900
 225

 1237
 38

 3

 3

 18

 1198
 38

 517
 8

 18

 18

 214
 42

 25

 4

 3

 18

 189
 42

 2

 11

 103
 18

 71
 21

 7

 4

 3

 3

 27
 4

 27
 4

 523
 46

 523
 46

 2

 521
 46

 1846
 74

 426
 33

 1420
 41

 1420
 41

 31

 31

 31

 31

 475
 4

 112

 112

 112

 112

 363
 4

 363
 4

 25

 16

 9

 122

 122

 11

 3

 8

 15

 15

 15

 15

 15

 190
 241

 153
 195

 153
 195

 27
 156

 24
 156

 0
 41

 0
 41

 0
 41

 0
 41

 53
 36

 16
 33

 16
 33

 16
 33

 16
 33

 3

 4

 3

 0
 11

 0
 22

 13

 9

 9

 9

 9

 9

 4

 4

 4

 4

 4

 12
 136

 12
 126

 12
 126

 0
 10

 0
 10

 9
 14

 7

 7

 7

 7
