# Supplementary material for: Fungi are more transient than bacteria in caterpillar gut microbiomes
Source: Sci Rep. 2022 Sep 16;12:15552. doi: 10.1038/s41598-022-19855-5 (PMC9481635; doi:10.1038/s41598-022-19855-5)
Supplement: Supplementary file 3 — Supplementary Information. [file 41598_2022_19855_MOESM3_ESM.docx]

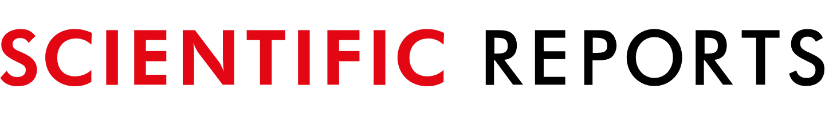


**Supplementary Information for**

Fungi are more transient than bacteria in caterpillar gut microbiomes

Martin Šigut, Petr Pyszko, Hana Šigutová, Denis Višňovská, Martin Kostovčík, Nela Kotásková, Ondřej Dorňák, Miroslav Kolařík and Pavel Drozd

Corresponding author: Martin Šigut

Email: [martin.sigut@osu.cz](mailto:xxxxx@xxxx.xxx)

**This file includes:**

Figures S3 to S8

Tables S2 and S3

Legends for Figures S1 and S2

Legends for Tables S1, S4 and S5

**Other supplementary materials for this manuscript include the following:**

Figures S1 and S2

Tables S1, S4 and S5


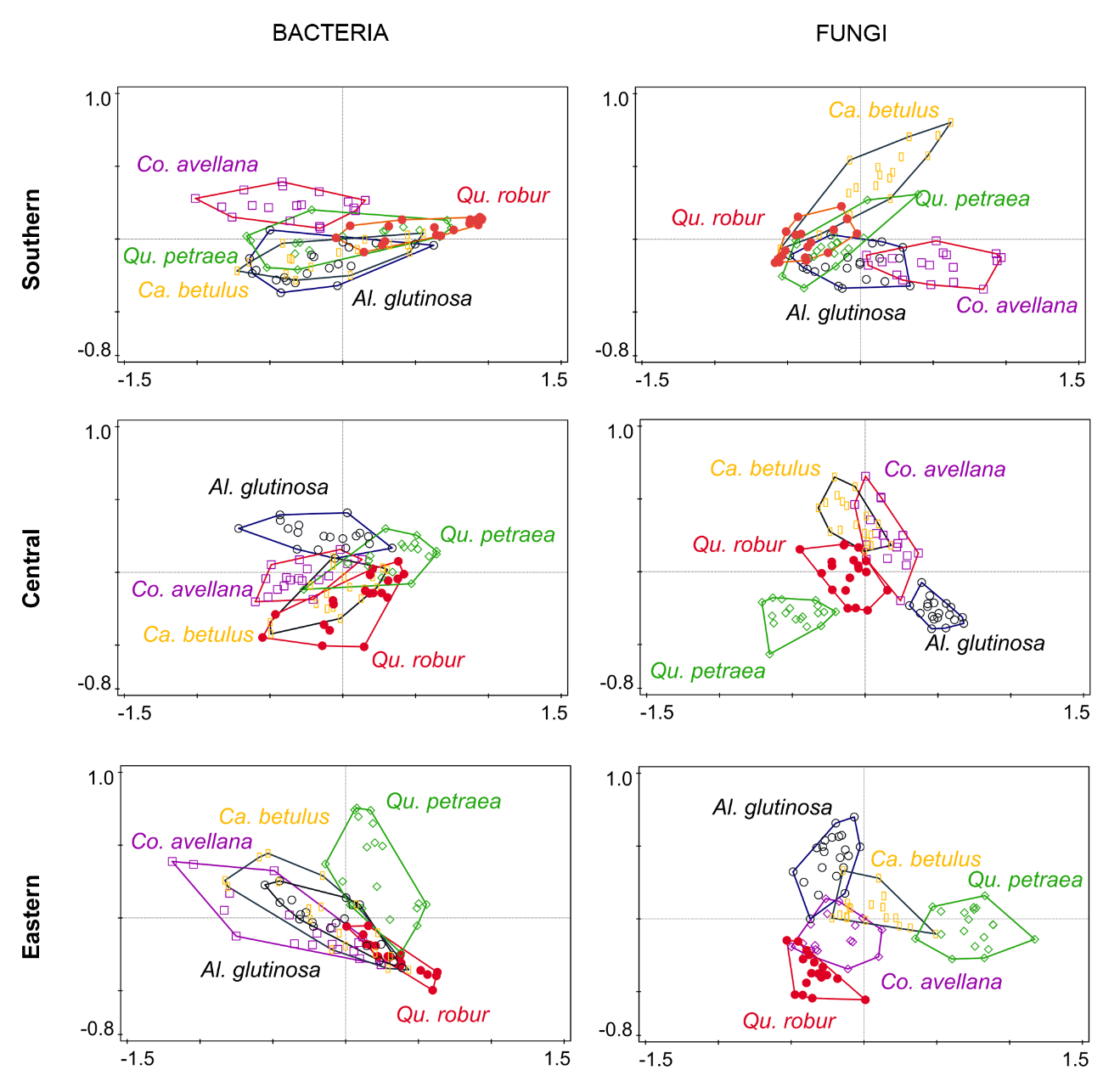


Fig. S3. Partial principal coordinates analysis (p-PCoA) plots of bacterial and fungal microbiota composition of leaves of individual host tree species (*Alnus glutinosa, Corylus avellana*, *Carpinus betulus*, *Quercus petraea* and *Q. robur*) on individual localities (Southern, Central and Eastern). The sampling plots were used as coordinates; each analysis was tested by Monte-Carlo test with 999 permutations. The differences in microbiota composition among individual tree species were always significant (*p* = 0.001).


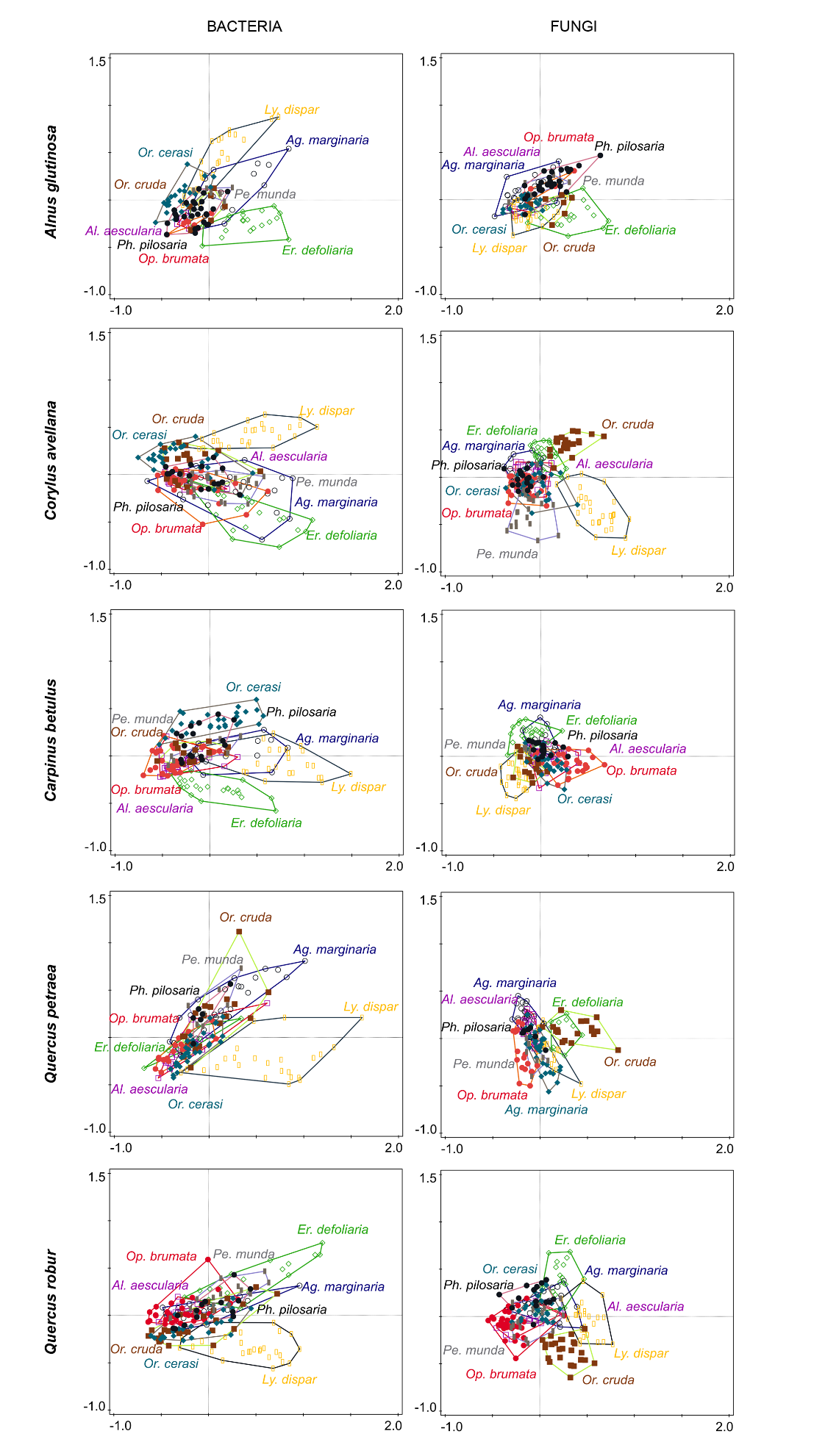


Fig. S4. Partial principal coordinates analysis (p-PCoA) plots of bacterial and fungal microbiota composition of individual polyphagous caterpillar species (*Agriopis marginaria*, *Operophtera brumata*, *Alsoplia aescularia*, *Erannis defoliaria*, *Phigalia pilosaria*, *Perigrapha munda*, *Orthosia cerasi*, *O. cruda*, *Lymantria dispar*) at host tree species (*Alnus glutinosa, Corylus avellana*, *Carpinus betulus*, *Quercus petraea* and *Q. robur*). The localities were used as coordinates; each analysis was tested by Monte-Carlo test with 999 permutations. The differences in microbiota composition among individual tree species were always significant (*p* = 0.001). For fungal microbiota on *C. avellana* and *Q. robur*, rare species were downweighted before analysis.


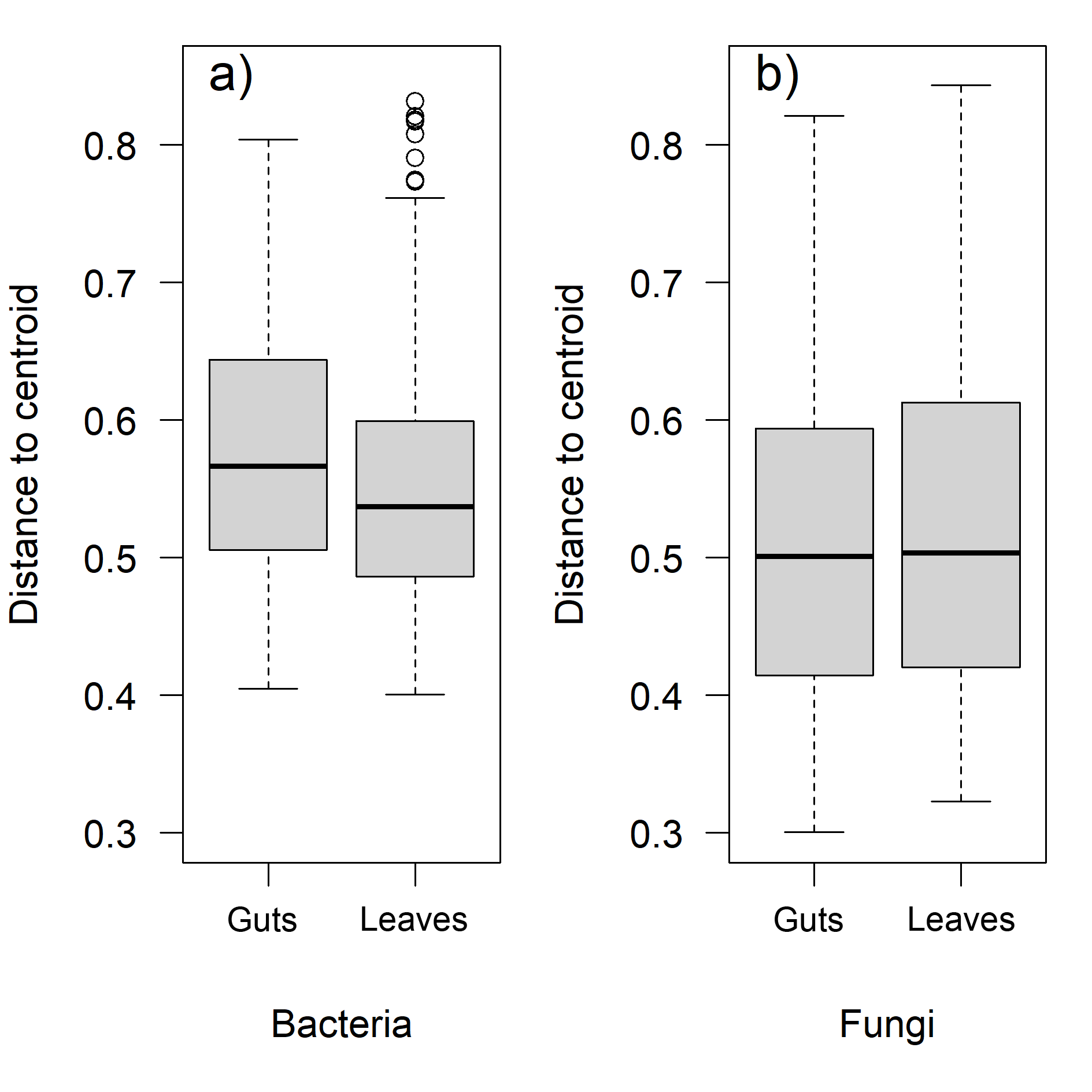


Fig. S5. The dispersion in β-diversity of samples of gut and leaf microbiota measured as distance to centroid (Bray-Curtis distance) for a) bacterial and b) fungal dataset.


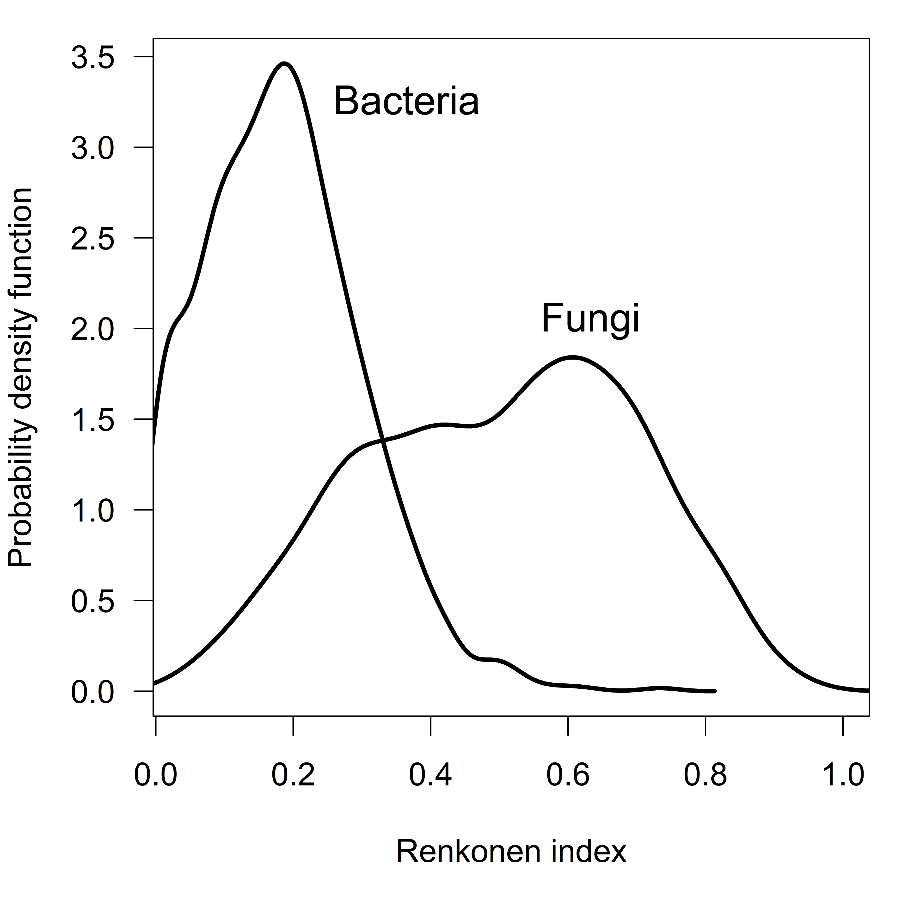


Fig. S6. Comparison of distribution of Renkonen similarity index between caterpillar and leaf samples for bacteria and fungi.


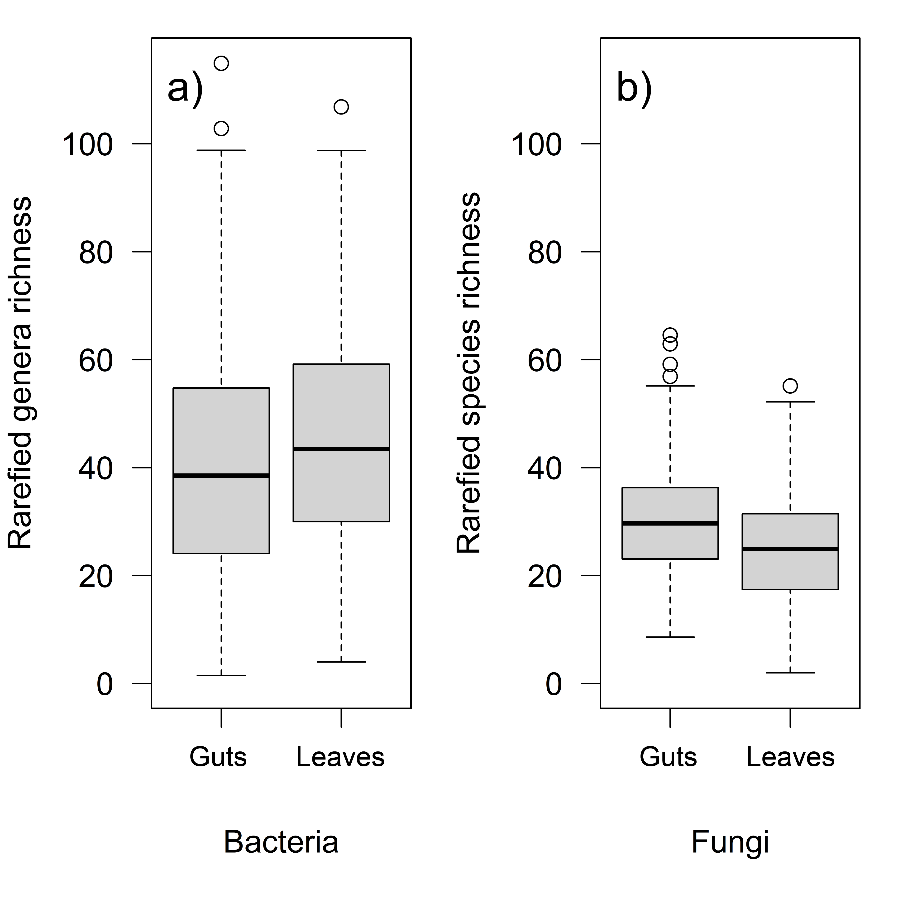


Fig. S7. Comparison of rarefied a) bacterial genera richness and b) fungal species richness between caterpillar gut and leaf samples. The number of reads in each sample was rarefied to 400.


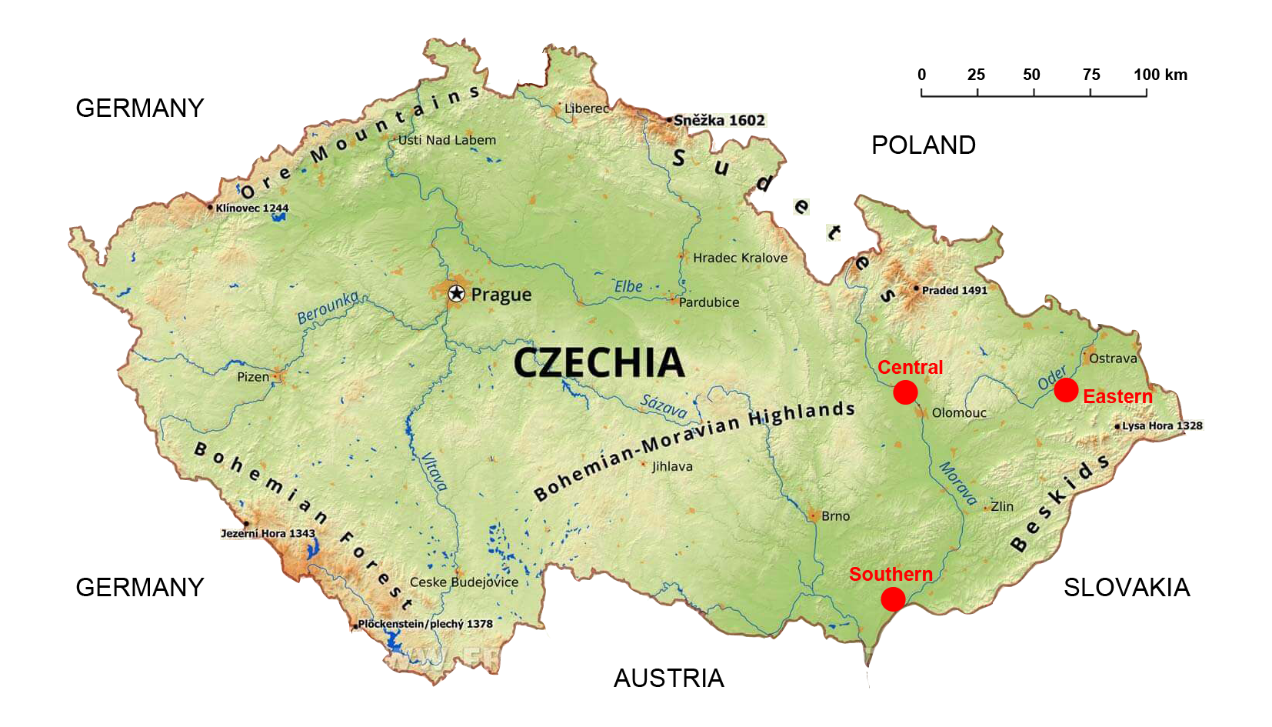


Fig. S8. Map of the Czechia showing three sampling localities (in red): Southern Moravia (Hodonínská Dúbrava), Central Moravia (Střeň in PLA Litovelské Pomoraví) and Eastern Moravia (PLA Poodří). The map backgroud was taken from the website FreeWorldMaps.net (<https://www.freeworldmaps.net/europe/czechia/czechia-physical-map.jpg>).

Table S2. Number of caterpillar and host tree species sampled at individual localities.

| **Organism** | | | | **Locality** | | | |
| --- | --- | --- | --- | --- | --- | --- | --- |
| **Type** | **Order** | **Family** | **Species** | **Southern** | **Central** | **Eastern** | **Total** |
| caterpillar | Lepidoptera | Erebidae | *Lymantria dispar* (Linnaeus, 1758) | 45 | 45 | 39 | 129 |
| caterpillar | Lepidoptera | Geometridae | *Agriopis marginaria* (Fabricius, 1776) | 30 | 27 | 45 | 102 |
| caterpillar | Lepidoptera | Geometridae | *Alsophila aescularia* (Denis & Schiffermüller, 1775) | 4 | 16 | 61 | 81 |
| caterpillar | Lepidoptera | Geometridae | *Erannis defoliaria* (Clerck, 1759) | 43 | 39 | 12 | 94 |
| caterpillar | Lepidoptera | Geometridae | *Operophtera brumata* (Linnaeus, 1758) | 45 | 48 | 22 | 115 |
| caterpillar | Lepidoptera | Geometridae | *Phigalia pilosaria* (Denis & Schiffermüller, 1775) | 23 | 28 | 28 | 79 |
| caterpillar | Lepidoptera | Noctuidae | *Orthosia cerasi* (Fabricius, 1775) | 23 | 58 | 56 | 137 |
| caterpillar | Lepidoptera | Noctuidae | *Orthosia cruda* (Denis & Schiffermüller, 1775) | 23 | 41 | 24 | 88 |
| caterpillar | Lepidoptera | Noctuidae | *Perigrapha munda* (Denis & Schiffermüller, 1775) | 23 | 27 | 8 | 58 |
| tree | Fagales | Betulaceae | *Alnus glutinosa* (L.) Gaertn. | 21 | 19 | 21 | 61 |
| tree | Fagales | Betulaceae | *Carpinus betulus* L. | 18 | 18 | 18 | 54 |
| tree | Fagales | Betulaceae | *Corylus avellana* L. | 18 | 18 | 18 | 54 |
| tree | Fagales | Fagaceae | *Quercus petraea* (Matt.) Liebl*.* | 18 | 18 | 19 | 55 |
| tree | Fagales | Fagaceae | *Quercus robur* L. | 18 | 19 | 18 | 55 |
| **Total** | | | | 352 | 421 | 389 | 1162 |

Table S3. List of taxonomic resources used for identification of caterpillars.

1. Laštůvka, Z. & Liška, J. (2010) Checklist of Lepidoptera of the Czech Republic (Insecta: Lepidoptera). URL http://lepidoptera.wz.cz.
2. Macek, J., Dvořák, J., Traxler, L. & Červenka, V. (2007) Noční motýli I. Academia, Praha.
3. Macek, J., Dvořák, J., Traxler, L. & Červenka, V. (2008) Noční motýli II. Academia, Praha.
4. Macek, J., Procházka, J. & Traxler, L. (2012) Noční motýli III. Academia, Praha.
5. Patočka, J. (1980) Die Raupen und Puppen der Eichenschmetterlinge Mitteleuropas. Monographien zur angewandten Entomologie.
6. Ratnasingham, S. & Hebert, P. D. N. (2007) BOLD: The Barcode of Life Data System. *Molecular Ecology Notes* 7, 355–364. URL http://barcodinglife.org.

Figure S1 (separate file). Krona chart showing bacterial composition of leaf and gut microbiota at different taxonomic levels recovered by 16S DNA metabarcoding. The width of each sector corresponds to the relative proportion of its reads. Taxonomic levels are displayed hierarchically from phyllum (the innermost layer) to genus level (the outermost layer). Rickettsiales are excluded.

Figure S2 (separate file). Krona chart showing fungal composition of leaf and gut microbiota at different taxonomic levels recovered by ITS2 DNA metabarcoding. The width of each sector corresponds to the relative proportion of its reads. Taxonomic levels are displayed hierarchically from phyllum (the innermost layer) to species level (the outermost layer).

Table S1 (separate file). Indicator bacterial and fungal taxa significantly associated with leaves and guts, and individual caterpillar and tree species.

Table S4 (separate file). Bacterial and fungal ASVs with the respective number of contaminant reads identified by library “decontam” in host tree leaves and caterpillar guts.

Table S5 (separate file). Overview of leaf and gut samples with number of reads of recovered bacterial and fungal taxa along with variables used for analyses of composition and richness of leaf and gut microbiota.
